# Plastid-localized xanthorhodopsin increases diatom biomass and ecosystem productivity in iron-limited surface oceans

**DOI:** 10.1038/s41564-023-01498-5

**Published:** 2023-10-16

**Authors:** Jan Strauss, Longji Deng, Shiqiang Gao, Andrew Toseland, Charles Bachy, Chong Zhang, Amy Kirkham, Amanda Hopes, Robert Utting, Eike F. Joest, Alessandro Tagliabue, Christian Löw, Alexandra Z. Worden, Georg Nagel, Thomas Mock

**Affiliations:** 1 School of Environmental Sciences, University of East Anglia, Norwich Research Park, Norwich, UK; 2 Ocean EcoSystems Biology Unit, RD3, GEOMAR Helmholtz Centre for Ocean Research Kiel, Kiel, Germany; 3 European Molecular Biology Laboratory (EMBL), Hamburg Unit c/o Deutsches Elektronen Synchrotron (DESY), Hamburg, Germany; 4 Centre for Structural Systems Biology (CSSB), Hamburg, Germany; 5 Department of Neurophysiology, Institute of Physiology, University of Würzburg, Wuerzburg, Germany; 6 Sorbonne Université, CNRS, FR2424, Station biologique de Roscoff, Roscoff, France; 7 Department of Biology, Biocenter, University of Würzburg, Wuerzburg, Germany; 8 School of Environmental Sciences, University of Liverpool, Liverpool, UK; 9 Max Planck Institute for Evolutionary Biology, Plön, Germany; 10 Marine Biological Laboratory, Woods Hole, MA USA; 11 Present Address: German Maritime Centre, Hamburg, Germany; 12 Present Address: School of Biological Sciences, University of East Anglia, Norwich Research Park, Norwich, UK

**Keywords:** Water microbiology, Enzyme mechanisms

## Abstract

Microbial rhodopsins are photoreceptor proteins that convert light into biological signals or energy. Proteins of the xanthorhodopsin family are common in eukaryotic photosynthetic plankton including diatoms. However, their biological role in these organisms remains elusive. Here we report on a xanthorhodopsin variant (*Fc*R1) isolated from the polar diatom *Fragilariopsis cylindrus*. Applying a combination of biophysical, biochemical and reverse genetics approaches, we demonstrate that *Fc*R1 is a plastid-localized proton pump which binds the chromophore retinal and is activated by green light. Enhanced growth of a *Thalassiora pseudonana* gain-of-function mutant expressing *Fc*R1 under iron limitation shows that the xanthorhodopsin proton pump supports growth when chlorophyll-based photosynthesis is iron-limited. The abundance of xanthorhodopsin transcripts in natural diatom communities of the surface oceans is anticorrelated with the availability of dissolved iron. Thus, we propose that these proton pumps convey a fitness advantage in regions where phytoplankton growth is limited by the availability of dissolved iron.

## Abstract (web summary)

The polar diatom *Fragilariopsis cylindrus* can circumvent limited chlorophyll-based photosynthesis by using a rhodopsin proton pump to fuel growth.

## Main

Microorganisms can convert sunlight into biological signals or metabolic energy using rhodopsins^1–5^. Microbial (type 1) rhodopsins are integral membrane photoreceptor proteins with seven or eight transmembrane helices^6–8^ that bind a β-carotene-derived retinal chromophore^1–3,9^. They are an abundant family of light-harvesting proteins^10^ and comprise a large diversity that is found in all domains of life^5,11^. Most microbial rhodopsins appear to bind all-*trans*-retinal to absorb light in the wavelengths of ~400–600 nm and catalyse a wide range of different biological functions^3,6,12–15^.

Since the discovery of the light-driven proton pumps bacteriorhodopsin in the archaeon *Halobacterium salinarum*^16^ and proteorhodopsin from an uncultured marine Gammaproteobacterium^17^, genomic and metagenomic analyses have revealed a wealth of rhodopsins from microbes ranging from Asgard archaea^18^ to eukaryotic microorganisms, including all major algal groups and even giant viruses^19–23^. Consequently, microbial rhodopsins are proposed to be major marine light capturers and estimates suggest they may absorb as much light as chlorophyll-based photosynthesis in the sea^24,25^.

The first eukaryotic microbial rhodopsins with proven proton-pumping functions were discovered in the fungal pathogen *Leptosphaeria maculans*^26^ and in the marine green alga *Acetabularia acetabulum*^27^ and ascribed to fungal and algal proton pump families with bacteriorhodopsin-like DTD motifs, respectively^2^. Subsequent work identified a substantial number of distantly related proton pumps from the xanthorhodopsin family in other marine algae such as dinoflagellates^20,28–30^, haptophytes^19^ and diatoms^19,31–33^. Moreover, recent structural characterization of fungal rhodopsins suggests that archaeal and eukaryotic proton pumps share a common ancestor^34^. However, their biological role in eukaryotes, including phytoplankton, has remained elusive.

Over large parts of the ocean, growth of eukaryotic phytoplankton is regulated by the micronutrient iron^35^, with the majority of the cellular iron demand being associated with photosynthesis^36^. To cope with iron limitation, phytoplankton have evolved a range of iron-sparing mechanisms, including replacement of iron in various catalysts^37,38^, acquisition of multiple forms of iron and modification of their photosynthetic architecture^39–41^. Resolving these mechanisms and their prevalence across different oceanic regions is critical to understanding how changes in iron availability impact phytoplankton growth and therefore ecosystem productivity.

Iron limitation has been proposed to induce expression of xanthorhodopsins, on the basis of preliminary results from gene expression studies using cultured phytoplankton and metatranscriptomes from natural communities^42,19,32,43^. Chlorophyll-based photosynthesis is limited by iron availability, thus it was suggested that xanthorhodopsins provide a selective advantage under these conditions^44^. Accordingly, rhodopsin proton pumps identified in diatoms have been proposed to play a light-harvesting role leading to the production of adenosine triphosphate (ATP) when iron availability limits chlorophyll-based photosynthesis^32,43^. Here we critically examine this hypothesis by applying an integrative approach to study the function of *Fc*R1, a xanthorhodopsin gene copy variant from the cold-adapted diatom *Fragilariopsis cylindrus*. Expression in *Xenopus* oocytes allowed us to investigate electrophysiological characteristics of *Fc*R1, while recombinant expression of *Fc*R1 in the heterologous diatom host *Thalassiosira pseudonana*, which does not encode rhodopsin, coupled with bioassay experiments provided evidence that xanthorhodopsins support diatom growth under iron limitation. Furthermore, relative expression of xanthorhodopsin-encoding genes from surface ocean eukaryotic phytoplankton communities was found to correlate with a nutrient limitation index indicating iron limitation. Taken together, our results suggest that xanthorhodopsins support primary productivity contributions of algae in ~35% of the surface ocean where phytoplankton growth is limited by dissolved iron availability.

## Results

### Xanthorhodopsin gene copy variants in *F. cylindrus*

**Fig. 1:**
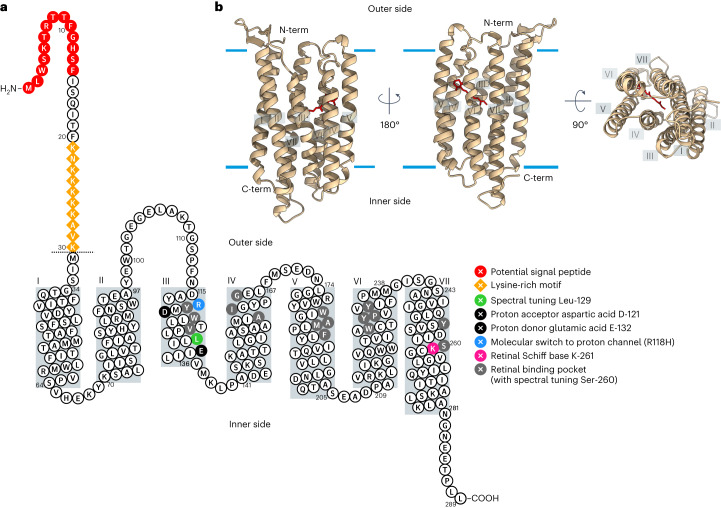
Identification and in silico analysis of *F. cylindrus* rhodopsin 1 (*Fc*R1). **a**, Secondary structure prediction of *Fc*R1 with key residues highlighted: proton acceptor D-121 and donor E-132 (black), K-261 (pink) forming a Schiff base link with retinal, and retinal binding pocket (Y-119, W-122, V-126, L-129, A-160, I-161, G-165, W-181, A-184, M-185, F-188, W-226, Y-229, P-230, Y-233, Y-253 and S-260) (light grey). The N-terminal extension of 30 amino acids of *Fc*R1 compared to *Fc*R2 is indicated as a dotted line. Potential N-terminal chloroplast signal peptide with conserved ‘ASAFAP’ (red circles) and lysine-rich motif (orange diamonds) in the N terminus of *Fc*R1 are highlighted. Grey boxes correspond to transmembrane helices as predicted by AlphaFold2 structure prediction in **b**. **b**, Overall 3D view of the *Fc*R1 protomer in the membrane as predicted by AlphaFold2. The experimental structure of PDB entry 4HYJ^42^ was used for structural alignment with *Fc*R1 to position the retinal ligand, and the final model was generated by removing the structure of PDB entry 4HYJ. Estimated hydrophobic–hydrophilic membrane boundaries are shown as light blue lines. Source data

The proportion of nucleotides that differ between both gene copy variants (*Fc*R1/2) of xanthorhodopsin in *F. cylindrus* was estimated to be 5.8%. Although their amino acid sequence is 100% identical, *Fc*R1 has an N-terminal extension of 30 amino acids, including a potential signal peptide that is cleaved in the mature protein. Full-length alignments of *Fc*R1/2 with characterized rhodopsins showed conserved residues responsible for a proton pump. The characteristic lysine residue (K-261) provides the retinal Schiff base linkage and acidic residues are found at positions of the proton acceptor (D-121) and donor (E-132) sites (Fig. 1a) homologous to K-216, D-85 and D-96 in the prototypic archaeal rhodopsin proton pump, bacteriorhodopsin (BR). AlphaFold2-based three-dimensional (3D) structure prediction^45^ (Fig. 1b) confirmed that *Fc*R1/2 consists of seven transmembrane helices with an antiparallel beta sheet and extended extracellular loop between helix II and helix III (Fig. 1a,b) as shown for fungal rhodopsins^34,46^, and high structural similarity to a rhodopsin from the extremophile permafrost bacterium *Exiguobacterium sibiricum*^42^ based on a structure homology search^47^.

**Fig. 2:**
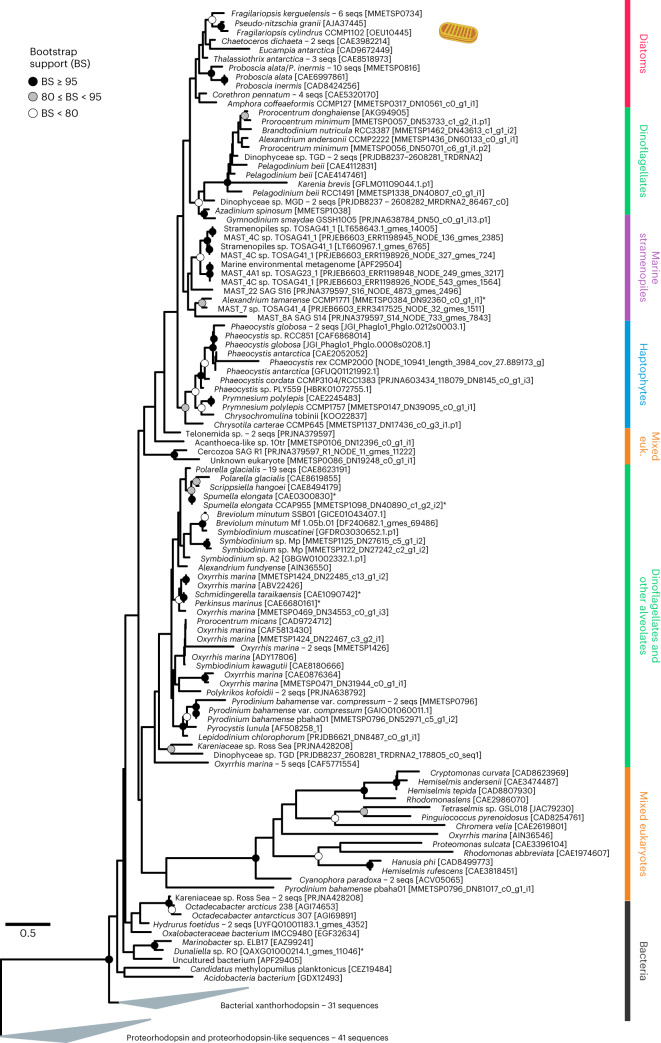
Phylogenetic relationships of *F. cylindrus* rhodopsin 1 (*Fc*R1) with eukaryotic rhodopsin protein sequences based on maximum likelihood. Broad taxonomic classification of rhodopsin sequences is indicated and the *Fc*R1 sequence is highlighted with an *F. cylindrus* icon. Nodes with multiple rhodopsin sequences of the same taxon were collapsed and accessions of the longest sequence are shown together with numbers of collapsed sequences. The tree is rooted in the proteorhodopsin clade for display purposes. Asterisks denote potentially falsely-annotated rhodopsin sequences in the database due to culture contamination. (Credit for *F*. *cylindrus* icon to Charlotte Eckmann.). Source data

**Extended Data Fig. 1:**
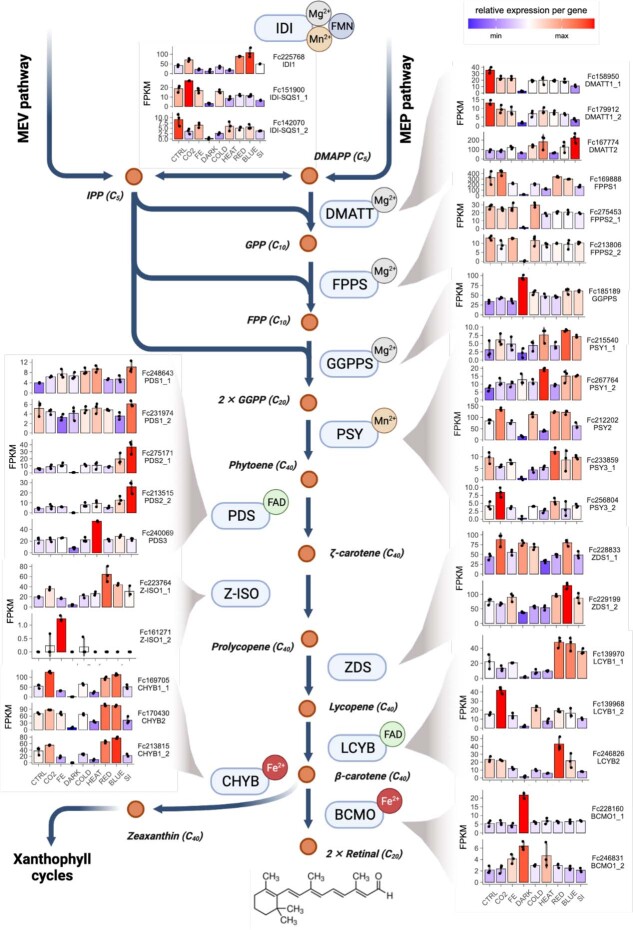
Natural retinal biosynthetic pathway from isoprenoid precursors in *Fragilariopsis cylindrus*. Bar charts show RNA-seq gene expression of genes involved in putative biosynthetic pathway of all-*trans*-retinal in *Fragilariopsis cylindrus* including the cytosolic MEV and plastid DXP/MEP (grey box) during nine replicated experimental conditions (n = 3). Color scale represents absolute gene expression values (FPKM) on a relative scale per gene/gene copy. Gene expression barcharts for identified enzymes and isoenzymes are shown together with Joint Genome Institute (JGI) protein identifiers. Data are presented as mean values +/- s.d. To distinguish gene models belonging to divergent allelic pairs encoding for a single enzyme, they are labelled with suffixes (‘_1’ and ‘_2’). Chemical compounds are represented as circles (red) and enzymes are represented as rounded rectangles (light blue) together with their essential cofactors. Abbreviations: BCMO, β-carotene 15,15’-monooxygenase; CHYB, β-carotene 3-hydroxylase; DMAPP, dimethylallyl diphosphate; DMATT, dimethylallyltranstransferase; FAD, flavin adenine dinucleotide; Fe^2^^+^ , ferrous iron; FMN, flavin mononucleotide; FPKM, fragments per kilobase of exon per million mapped fragments; FPP, farnesyl diphosphate; FPPS, farnesyl diphosphate synthase; GGPP, geranylgeranyl diphosphate; GGPPS, geranylgeranyl diphosphate synthase; GPP, geranyl diphosphate; IDI, IPP:DMAPP isomerase; IPP, isopentenyl diphosphate; LCYB, lycopene β-cyclase; MEP, Methylerythritol phosphate; MEV, mevalonic acid; Mg^2^^+^ , divalent magnesium ion; Mn^2^^+^ , divalent manganese ion; PDS, phytoene desaturase; PSY, phytoene synthase; ZDS, ζ-carotene desaturase; Z-ISO, ζ-carotene isomerase. Source data

The essential retinal chromophore for microbial opsins can be provided via a putative biosynthetic pathway including a β-carotene cleavage enzyme (β-carotene 15,15’-dioxygenase, EC 1.13.11.63), of which we annotated two putative isoforms in the *F. cylindrus* genome sequence (BCMO1_1 JGI ID: 228160, Uniprot: A0A1E7EZR0, GenBank: OEU11319; BCMO1_2 JGI ID: 246831) and also in the sequenced diatoms *Phaeodactylum tricornutum* (Uniprot: B7G4J2) and *T. pseudonana* (Uniprot: B8C6K5). Additional light-harvesting antennas as shown for fenestrated xantho- and proteorhodopsins^48,49^ may further be provided by competing carotenoid biosynthetic and xanthophyll cycle enzymes. Using RNA-seq transcriptome analysis, we showed that the biosynthetic genes were significantly expressed over an array of nine experimental conditions (Extended Data Fig. 1). A phylogenetic analysis focusing on eukaryotic microbial rhodopsins confirmed that *Fc*R1 belongs to the xanthorhodopsin family (Fig. 2). The phylogenetic tree further showed that eukaryotic rhodopsins generally followed the current established evolution of the group, but also gave evidence of horizontal gene transfer. Diatom, haptophyte and uncultured marine stramenopiles rhodopsins formed coherent monophyletic clades, whereas dinoflagellate rhodopsins appeared paraphyletic with two main clusters (Fig. 2).

**Fig. 3:**
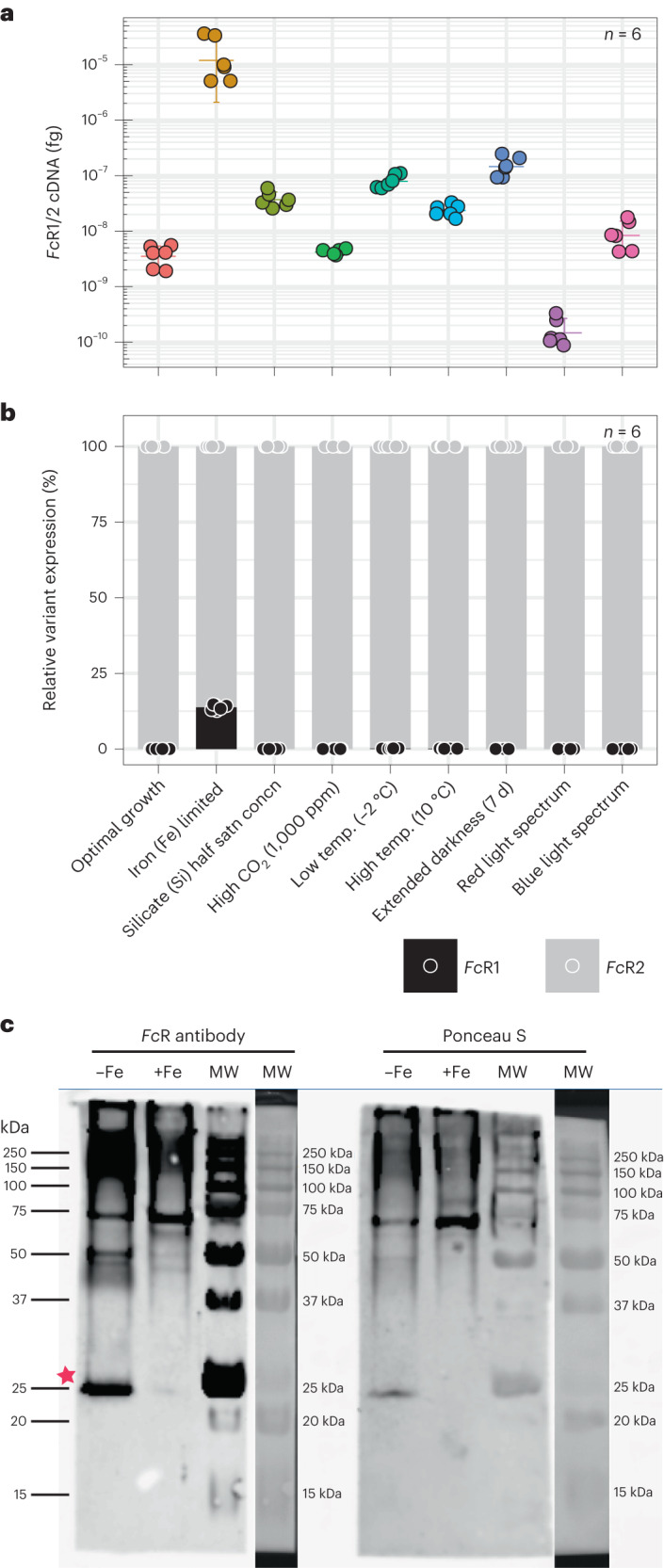
Gene and protein expression of *F. cylindrus* rhodopsin (*Fc*R) during iron-limited growth. **a**, Absolute RT–qPCR gene transcript quantification of *Fc*R1/2 under nine different growth conditions. Statistics are displayed as mean ± s.d. (*n* = 6) and individual datapoints are colour-coded by condition. **b**, Variant-specific RT–qPCR gene expression analysis of *Fc*R1 and *Fc*R2 under the same nine growth conditions as in **a**. Relative variant expression of total *Fc*R expression is shown. Statistics are displayed as mean ± s.d. (*n* = 6) and individual datapoints are colour-coded by variant. **c**, Representative western blot analysis of *Fc*R1/2 protein levels in iron-limited (−Fe) and iron-replete (+Fe) *F. cylindrus* using a custom *Fc*R antibody from three independent experiments. The star indicates the *Fc*R1/2 protein band with a predicted molecular weight between 29–32 kDa. Reversible Ponceau S staining before antibody probing was used as a loading control (right side). Molecular weight (MW) marker lanes are shown in different illumination modes. Note that probably due to *Fc*R1 and *Fc*R2 proteins having similar size after cleavage of the putative *Fc*R1 N-terminal signal peptide (Fig. 1a), separate bands were not resolved for both proteins. Source data

**Extended Data Fig. 2:**
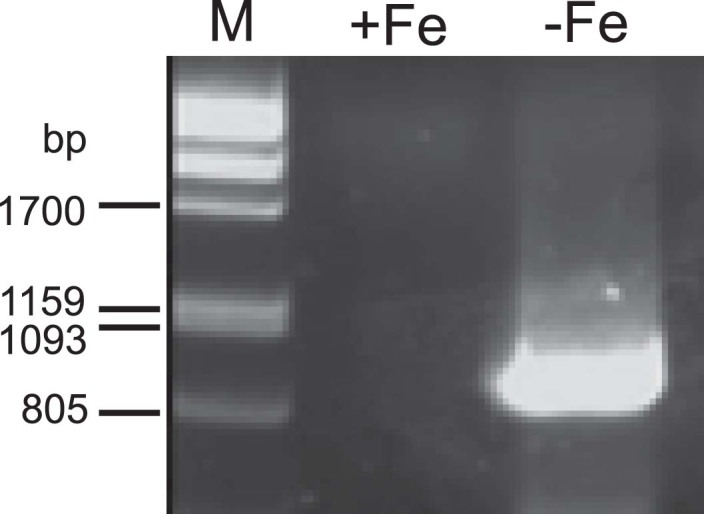
Amplification of *Fc*R1 from cDNA template. (a) Molecular cloning of *Fc*R1 from *F. cylindrus* cDNA generated from cell cultures grown under low iron. +Fe, *F. cylindrus* cDNA from nutrient replete cell cultures, -Fe, *F. cylindrus* cDNA from iron-limited cell cultures. A representative gel of three independent experiments is shown. Source data

The absolute quantification of both *Fc*R gene copies (*Fc*R1/2) showed a higher gene expression (by more than two orders of magnitude) in iron-limited *F. cylindrus* when their expression levels were combined (Fig. 3a). However, *Fc*R1 was only (and strongly) induced during iron-limited growth, whereas *Fc*R2 was detected during all tested conditions (Fig. 3b). In agreement with quantitative PCR with reverse transcription (RT–qPCR) results, full-length *Fc*R1 complementary DNA (cDNA) transcripts could only be amplified from *F. cylindrus* grown under iron limitation (Extended Data Fig. 2), whereas *Fc*R2 could additionally also be amplified from other experimental conditions. The strong induction of the *Fc*R1 transcript under iron limitation was also reflected at the protein level (Fig. 3c). Note that probably due to the similar protein sizes between *Fc*R1 and *Fc*R2 after cleavage of the putative N-terminal signal peptide of *Fc*R1 (Fig. 1a), we were unable to resolve separate bands for both proteins. Protein expression confirmed the RT–qPCR results (Fig. 3c).

### Biophysical characterization of *Fc*R1/2

**Fig. 4:**
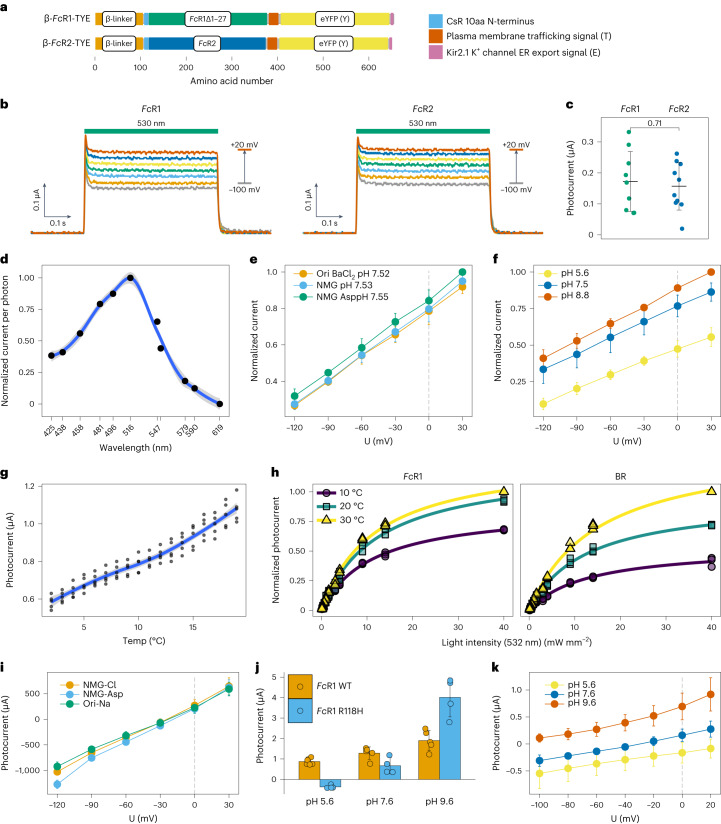
Biophysical characterization of *F. cylindrus* rhodopsin variants (*Fc*R1/2) as cold-adapted green light-driven proton pumps. **a**, Schematics of DNA constructs used for TEVC analysis in *X. laevis* oocytes. Orange, amino acids 1–105 of rat gastric H^+^/K^+^-ATPase β-subunit fragment (β-linker); sky blue, amino acids 1–10 of *C. subellipsoidea* rhodopsin (*Cs*R); bluish green, N-terminally truncated *Fc*R1 (*Fc*R1Δ27); blue, full-length *Fc*R2; vermillion, the plasma membrane trafficking signal from Kir2.1 K^+^ channel (T); yellow, enhanced yellow fluorescent protein (eYFP); reddish purple, the endoplasmic reticulum export signal from Kir2.1 K^+^ channel (E); grey, FLAG-tag (FLAG). **b**, Voltage dependence of the green light-induced pump signal of *Fc*R1 and *Fc*R2. Currents were recorded at incremental membrane potential steps of 20 mV from −100 mV (grey) to +20 mV (vermillion). A 530 nm laser was used for illumination. **c**, Comparison of *Fc*R1 and *Fc*R2 photocurrents. Statistics are displayed as mean ± s.d. (*n* = 10) and the *P* value displayed was determined by a two-sided unpaired *t*-test. **d**, Action spectrum of *Fc*R1. *Fc*R1 photocurrent was measured in NMG buffer with pH 7.5 at a membrane potential of −20 mV. The photocurrent was normalized to the *Fc*R1 photocurrent with 516 nm light illumination and is shown as arbitrary units. Statistics are displayed as mean ± s.d. (*n* = 3) and the blue line shows smoothed conditional means (loess smooth) of photocurrents, including their 0.95 confidence intervals (grey). **e**, *Fc*R1 photocurrents in Ori BaCl_2_ pH 7.5, NMG pH 7.5 and NMG Asp pH 7.55 buffers and at membrane potentials from −120 mV to +30 mV. The photocurrent was normalized to the *Fc*R1 photocurrent in NMG Asp pH 7.55 buffer at a membrane potential of +30 mV and is shown as arbitrary units. A 532 nm laser was used for illumination. Statistics are displayed as mean ± s.d. (*n* = 4). **f**, *Fc*R1 photocurrent measured in NMG buffer with pH 5.6, 7.5 and 8.8 at membrane potentials from −120 mV to +30 mV. The photocurrent was normalized to the *Fc*R1 photocurrent in NMG pH 8.8 buffer at a membrane potential of +30 mV and is shown as arbitrary units. A 532 nm laser was used for illumination. Statistics are displayed as mean ± s.d. (*n* = 3). **g**, *Fc*R1 photocurrents at different temperatures (*n* = 5). Oocytes were incubated in ND96 buffer containing 1 µM all-*trans-*retinal, and 532 nm green light was used for illumination. The blue line shows smoothed conditional means (loess smooth) of photocurrents, including their 0.95 confidence intervals (grey). **h**, Comparison of *Fc*R1 and BR photocurrents at different temperatures and light intensities. Photocurrents were tested at 10°C (ripe plum, circles), 20°C (bluish green, squares) and 30°C (light yellow, triangles) and different light intensities (*n* = 3). Oocytes were incubated in ND96 buffer containing 1 µM all-*trans*-retinal and 532 nm green light was used for illumination. Photocurrents were normalized and are shown as arbitrary units. **i**, Green-light-induced photocurrents of *Fc*R1 R118H mutant in three different extracellular buffers: NMG Asp pH 7.7, NMG pH 7.6 and Ori BaCl_2_ pH 7.6. Currents were recorded at incremental membrane potential steps of 30 mV from −120 mV to −30 mV. Statistics are displayed as mean ± s.d. (*n* = 5). **j**, Comparison of stationary photocurrents of *Fc*R1 wild type (WT, *n* = 5) and *Fc*R1 R118H (*n* = 4) in NMG buffers of different pHs at a membrane potential of 0 mV. Statistics are displayed as mean ± s.d. **k**, Green-light-induced photocurrents of *Fc*R1 R118H mutant in NMG buffers of different pHs. Currents were recorded at incremental membrane potential steps of 20 mV from −100 mV to +20 mV. Statistics are displayed as mean ± s.d. (*n* = 5). Source data

**Extended Data Fig. 3:**
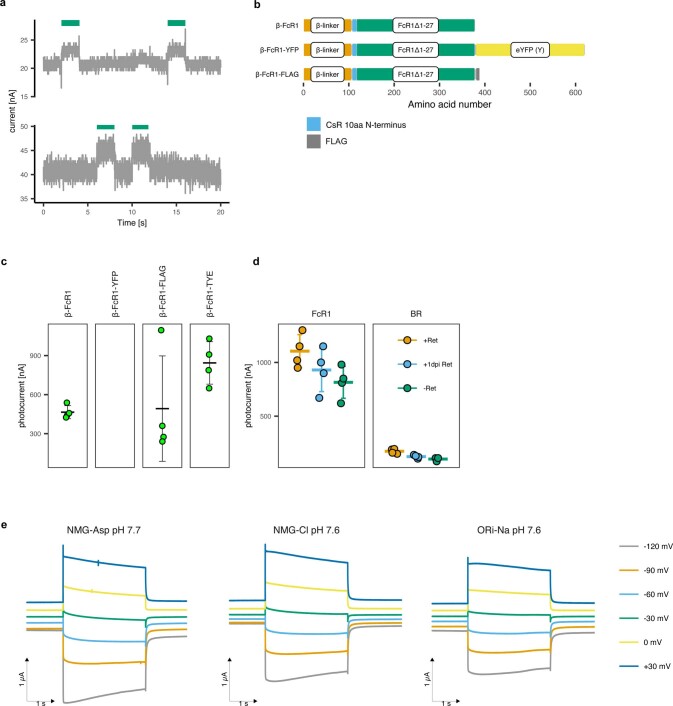
Biophysical characterization of *Fc*R1 and comparison to bacteriorhodopsin. (**a**) Photocurrent of *Fc*R1 wild-type without N and C terminal modifications measured using two-electrode voltage clamp in two *Xenopus laevis* oocytes. (**b**) Schematics of DNA constructs used for two-electrode voltage clamp analysis in *X*. *laevis* oocytes. Orange, amino acids 1–105 of rat gastric H^+^ /K^+^ -ATPase β-subunit fragment (β-linker); sky blue, amino acids 1–10 of *Coccomyxa* *subellipsoidea* rhodopsin (CsR); bluish green, N-terminally truncated *Fc*R1 (FcR1Δ27); yellow, enhanced yellow fluorescent protein (eYFP); gray, FLAG-tag (FLAG). (**c**) Photocurrents of different *Fc*R1 expression constructs. Statistics are displayed as mean ± s.d. (n = 4) together with individual data points. (**d**) *Fc*R1 and bacteriorhodopsin (BR) photocurrents with and without additional all-trans retinal (Ret). +Ret (orange), incubation of oocytes in ND96 buffer containing 1 µM Ret. –Ret (green), no additional Ret in ND96 buffer. +1dpi Ret (light blue), 1 µM Ret was added to ND96 buffer one day post oocyte injection (1 dpi). Photocurrent was measured at a membrane potential of -30 mV in Ori NMG pH 7.5 buffer. A 532 nm green light laser was used for illumination. Statistics are displayed as mean ± s.d. (n = 4). (**e**) Representative green light-induced photocurrent traces of *Fc*R1 R118H mutant in three different extracellular buffers: NMG Asp pH 7.7, NMG pH 7.6 and Ori BaCl2 pH 7.6. Currents were recorded at incremental membrane potential steps of 30 mV from -120 mV to +30 mV. Source data

*Fc*R1 and 2 were characterized and compared to the prototypic archaeal rhodopsin proton pump BR by expression in *Xenopus* oocytes. On the basis of the conserved residues and structural models, we expected both *Fc*Rs to act as proton pumps. Indeed, we observed photocurrents of up to 5 nA in some oocytes expressing *Fc*R1 wild type during initial two-electrode voltage clamp (TEVC) measurements (Extended Data Fig. 3a) but no photocurrents for *Fc*R2, thus we focused on optimization of *Fc*R1 expression in oocytes for reproducible biophysical characterization (Fig. 4a,b and Extended Data Fig. 3b,c).

Upon illumination with green or blue light, *Fc*R1/2 responded with a typical ion pump signal, showing no significant difference between the two proteins when applying the same optimized expression construct (Fig. 4b,c). Given the spectral tuning of microbial rhodopsins in the marine environment, we analysed the spectral range promoting maximal pump activity of *Fc*R1 by recording its action spectrum. *Fc*R1 showed a bell-shaped action spectrum (Fig. 4d), with maximal pumping activity in the green spectrum (excitation at ~516 nm). Interestingly, the photocurrents of *Fc*R1 were significantly higher than those of BR^50^ but showed a similar dependence on all-*trans*-retinal (Extended Data Fig. 3d).

To test whether *Fc*R1 solely pumps protons, we analysed photocurrents in *Xenopus* oocytes in buffers of the same pH (7.5) but containing different ions. The *Fc*R1 photocurrents were measured at different potentials in all three buffers, showing no difference (Fig. 4e). We also tested the pH dependency of the pump activity. *Fc*R1 showed the expected behaviour of an outward-directed proton pump, with lowest pumping activity at negative voltages in a pH 5.5 buffer and highest pumping activities at positive voltages in a pH 8.8 buffer (Fig. 4f). Together, our data illustrate that the *Fc*R1 photocurrent is solely caused by protons.

We further tested the activity of *Fc*R1 at low temperatures because of the psychrophilic nature of *F. cylindrus*. Photocurrents of *Fc*R1 at 2°C were reduced to about half of its photocurrent at 19°C (Fig. 4g). Reducing temperatures from 30°C to 10°C, the photocurrent of *Fc*R1 at 40 mW mm^−2^ was reduced to only 68.02% ± 0.01, whereas the photocurrent of BR was reduced to 41.25% ± 0.04 (Fig. 4h). In addition, simultaneous pairwise comparisons of the half-maximal photocurrents (*K*_0.5_) indicated that *K*_0.5_ did not vary for *Fc*R1 but decreased in response to decreasing temperatures for BR, indicating that *Fc*R1 photocurrents were less sensitive to a reduction in temperature, as expected for a cold-adapted enzyme.

During molecular cloning, we unintentionally recovered a point mutation of arginine (R) to histidine (H) in helix III at position 118 (R118H, Fig. 1a). Since this residue is conserved in different microbial rhodopsins and is known to be important for proton transfer, we kept the *Fc*R1 (R118H) mutant for analysis of its photocurrents. Interestingly, the R118H mutant behaves like a proton channel allowing bidirectional transport of protons (Fig. 4i–k and Extended Data Fig. 3e). It displayed comparable current amplitudes in buffers with different anions and cations at similar pH levels (Fig. 4i and Extended Data Fig. 3e). Under conditions of approximately pH 7.6, we observed an inward proton flux at more negative potentials for *Fc*R1 R118H (Fig. 4i–k), whereas *Fc*R1 WT only demonstrated outward proton transport upon illumination (Fig. 4e,f,j). The reverse potential of *Fc*R1 R118H was determined to be approximately −15 mV at pH 7.6 (Fig. 4i). In addition, the current amplitude and reverse potentials of *Fc*R1 R118H displayed pH dependence (Fig. 4k). Together, our findings show that FcR1 R118H functions as a light-gated H^+^ channel. The discovery of a light-gated, H^+^-specific channel holds potential for optogenetic applications aiming at pH manipulation. Building on these findings, we successfully engineered light-gated proton channels derived from fungal rhodopsins by homologous point mutations^46^.

### Characterization of *Fc*R1 using reverse genetics and phenotyping

**Fig. 5:**
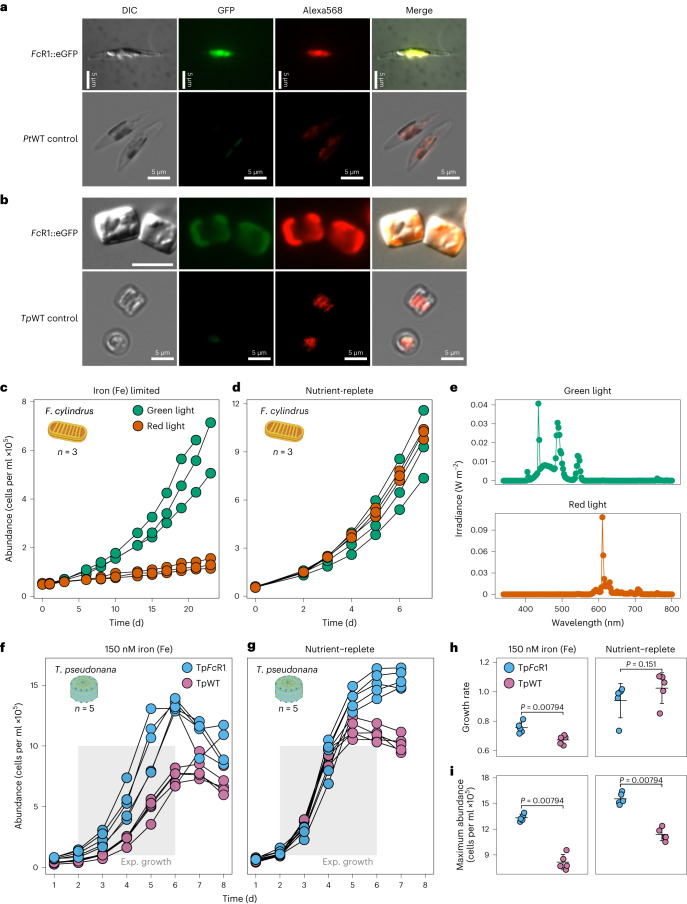
Subcellular localization of *Fc*R1 and phytoplankton phenotyping assays. **a**,**b**, Localization of *Fc*R1::eGFP fusion protein after expression in the pennate diatom *P. tricornutum* (**a**) and in the centric diatom *T. pseudonana* (**b**), including untransformed wild-type control strains (*Pt*WT and *Tp*WT, respectively). Shown are Normarski DIC, green fluorescence protein (GFP) and red chlorophyll autofluorescence (Alexa568). Representative micrographs of five independent experiments are shown. Scale bars, 5 µm. **c**, Growth of iron-limited *F. cylindrus* under green and red LEDs (*n* = 3). **d**, Growth of nutrient-replete *F. cylindrus* under green and red LEDs (*n* = 3). **e**, Spectral wavelength of green and red LEDs (*n* = 1). **f**, Growth of *T. pseudonana* rhodopsin knock-in gain-of-function (KI) mutant (Tp*Fc*R1, sky blue) and wild-type (TpWT, reddish purple) under iron limitation (150 nM) and constant white light (38 µmol photons m^−2^ s^−1^). Exponential growth phase to determine growth rates is highlighted as a grey-shaded box. **g**, Growth of *T. pseudonana* rhodopsin knock-in gain-of-function (KI) mutant (Tp*Fc*R1, sky blue) and wild type (TpWT, reddish purple) in nutrient-replete medium and constant white light (38 µmol photons m^−2^ s^−1^). Exponential growth phase to determine growth rates is highlighted as a grey-shaded box. **h**, Growth rates of *T. pseudonana* rhodopsin knock-in mutant (Tp*Fc*R1, sky blue) and *T. pseudonana* wild type (TpWT, reddish purple) under constant white light (38 µmol photons m^−2^ s^−1^) during iron-limited and nutrient-replete growth. **i**, Maximum cell abundance of *T. pseudonana* rhodopsin knock-in mutant (Tp*Fc*R1, sky blue) and *T. pseudonana* wild type (TpWT, reddish purple) under constant white light (38 µmol photons m^−2^ s^−1^) during nutrient-replete and iron-limited growth. Statistics are displayed as mean ± s.d. (*n* = 5) and the *P* values displayed were determined using two-sided Wilcoxon rank-sum test (**h**,**i**). (Credit for diatom illustrations of *F*. *cylindrus* (**c**,**d**) and *T*. *pseudonana* (**f**,**g**) to Charlotte Eckmann). Source data

**Extended Data Fig. 4:**
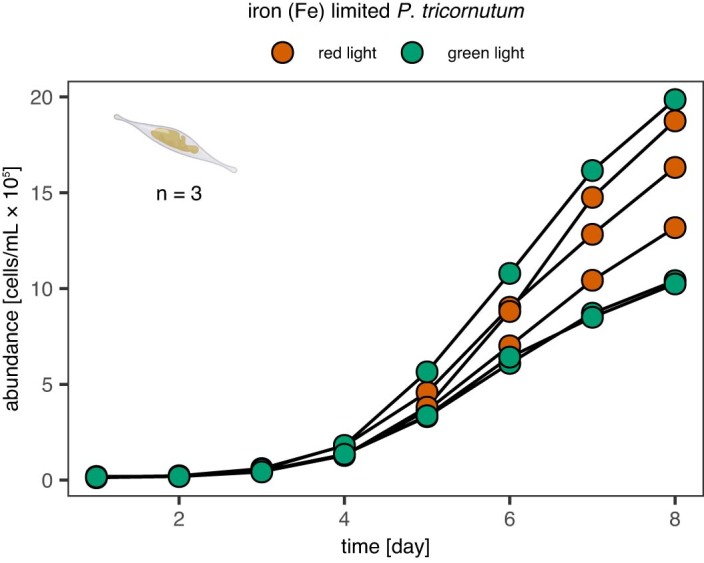
Iron-limited growth of diatom *Phaeodactylum tricornutum* wild-type strain UTEX646 under green and red light. Source data

To identify the subcellular localization of *Fc*R1, we expressed C-terminal eGFP fusion constructs of the full-length *Fc*R1 and *Fc*R1 N terminus (*Fc*R1::eGFP, *Fc*R1^N-term^::eGFP) in *P. tricornutum* and *T. pseudonana* using the biolistics method^51,52^. Using differential interference contrast (DIC) and epifluorescence microscopy, the fluorescence signals of *Fc*R1::eGFP and *Fc*R1^N-term^::eGFP transformants were observed to be overlapping with the chlorophyll fluorescence from the plastids of both species, suggesting *Fc*R1 subcellular localization in the plastid (Fig. 5a,b). These results, together with our gene expression data and previous studies, support the hypothesis that rhodopsins play a role in supporting algal growth when chlorophyll-based photosynthesis is iron-limited^32^. Furthermore, maximum activity of *Fc*R1 under green light indicates that growth benefits may be conveyed in this light spectrum. Thus, phenotyping experiments with *F. cylindrus* were conducted using a two-factor design: iron-limited and iron-replete growth at green and red light, each to test the interaction and effect of different light spectra and iron nutrition on growth. Iron-limited *F. cylindrus* showed significant growth differences under green versus red light, whereas no significant differences were observed for either green or red light under iron-replete growth (Fig. 5c–e). In comparison, using *P. tricornutum* as control, because it lacks rhodopsin, no difference in iron-limited growth under green or red light was observed (Extended Data Fig. 4). These results corroborate the hypothesis that the green light-driven *Fc*R1 proton pump facilitates growth when photosynthesis is iron-limited.

To provide additional evidence for *Fc*R1 being responsible for enhanced growth of diatoms under iron limitation, we generated a rhodopsin gain-of-function cell line using the model *T. pseudonana* because it lacks rhodopsin and, similar to *F. cylindrus*, is part of a broadly distributed genus. *T. pseudonana* was selected over *P. tricornutum* for testing the effect of recombinant expression of *Fc*R1 on growth of diatoms under iron limitation because it is less tolerant to iron limitation, making growth phenotyping at low iron concentrations more controllable. We cloned an untagged version of *Fc*R1 into *T. pseudonana* (Tp*Fc*R1) because tagging abolished its proton-pumping activity. We then used diagnostic PCR to verify its integration and expression. The Tp*Fc*R1^KI^ mutant grew faster under iron limitation and white light compared with the *T. pseudonana* wild type (*P* = 0.00794) and reached a higher biomass yield in stationary phase in both iron-limited and iron-replete media (*P* < 0.01) (Fig. 5f,g,i).

### Xanthorhodopsin in eukaryotic metatranscriptomes from pole-to-pole

**Fig. 6:**
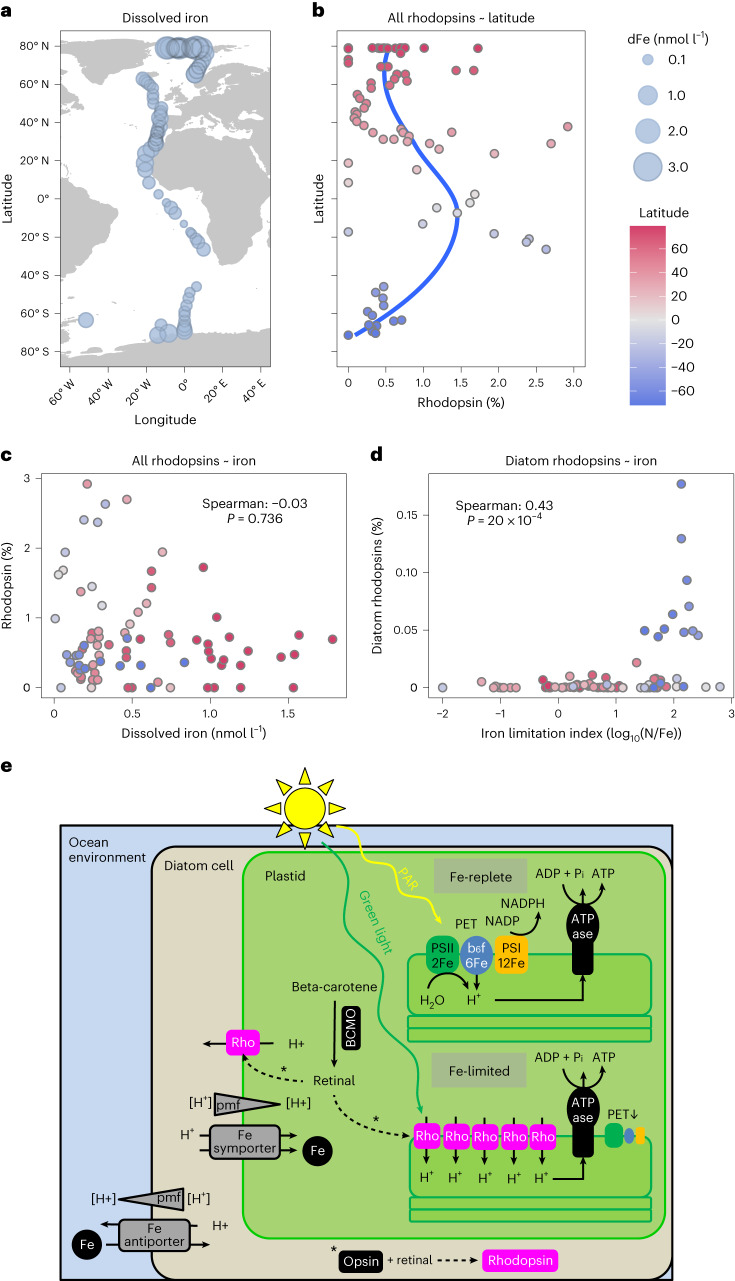
Environmental abundance of rhodopsin transcripts in metatranscriptomes and conceptual model of the physiological role of a diatom proton pump rhodopsin. **a**, Metatranscriptome sampling stations are scaled on the basis of estimated concentration of dissolved iron. **b**, Normalized environmental transcript abundances of eukaryotic rhodopsins as a function of latitude. The dark blue line shows smoothed conditional means (loess smooth) of rhodopsin transcript abundances. **c**, Correlation of normalized environmental rhodopsin transcript abundances and estimated dissolved iron concentration from a global ocean model. **d**, Correlation of estimated iron stress index and environmental diatom rhodopsin transcript abundances. The *P* values displayed were determined using a two-tailed *t*-test and Benjamini–Hochberg adjusted for multiple comparisons. **e**, Molecular physiological model of diatom rhodopsin. Source data

To evaluate the potential importance of proton-pumping xanthorhodopsins in supporting algal growth in iron-limited surface oceans, we performed a multiple regression analysis of the normalized abundance of xanthorhodopsin transcripts from 82 upper-ocean eukaryotic metatranscriptomes obtained from pole to pole^53,54^ with the environmental variables temperature, salinity, nitrate (N), phosphate (P), silicate (Si) and iron (Fe) (Fig. 6a). Missing values for observed N, P and Si concentrations were estimated from the World Ocean Atlas^55,56^ on the basis of sampling date, and concentrations of dissolved Fe were estimated from recent biogeochemical modelling efforts (Fig. 6b). Overall, the highest rhodopsin transcript abundances were observed at low latitudes (40° N–40° S) (Fig. 6b), often characterized by stratified upper-ocean waters with relatively low nutrient concentrations including dissolved iron.

**Extended Data Fig. 5:**
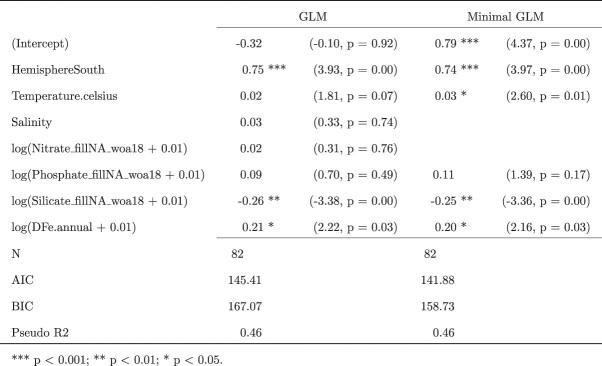
Generalized Linear Model results. We fitted a generalized linear model (estimated using ML) to predict rhodopsin abundance and derived a reduced minimal model based on Akaike Information Criterion (AIC) values using a backwards stepwise algorithm. Both models’ explanatory power is substantial (R2 = 0.40). Standardized parameters were obtained by fitting the models on a standardized version of the dataset. 95% Confidence Intervals (CIs) and p-values were computed using a Wald t-distribution approximation.

Application of a generalized linear model (GLM) showed that sampling location in the Southern Hemisphere explained most of the variance in eukaryotic microbial rhodopsin transcript abundances (*P* < 0.001), with Si (*P* = 0.001) and Fe (*P* = 0.029) being the nutrients significantly affecting eukaryotic microbial rhodopsin transcript abundances. A backwards stepwise algorithm to select variables from the initial GLM based on Akaike Information Criterion (AIC) values showed that sampling location in the Southern Hemisphere remained a significant factor for rhodopsin transcript abundances (*P* < 0.001), with both Si (*P* = 0.001) and Fe (*P* = 0.034) remaining significant. In addition, in the reduced minimal GLM, temperature appeared as a significant predictor of rhodopsin transcript abundances (*P* = 0.011) (Extended Data Fig. 5). Spearman’s rank correlations between eukaryotic rhodopsin transcript abundances and the selected environmental variables showed a positive correlation with temperature (*r*_(82)_ = 0.37, *P* = 0.001) and negative correlation with Si (*r*_(82)_ = −0.36, *P* = 0.001), but the relationship with Fe lacked statistical support (*r*_(82)_ = −0.04, *P* = 0.736).

Given the significance of silicate as a variable, and since diatoms differ from most other phytoplankton taxa in requiring silicic acid (Si(OH)_4_) to biomineralize their frustules, we reasoned that in addition to uncertainties in estimated environmental variables, our analysis may be confounded by including microbial rhodopsins from all eukaryotes, not just diatoms. We therefore used phylogenetic placement of metatranscriptome sequences onto a custom rhodopsin reference tree (Fig. 2) to identify putative diatom rhodopsins. A total of 58,316 translated rhodopsin metatranscripts were placed on the tree, of which 24,377 (41.8%) mapped to dinoflagellate rhodopsins and 3,439 (5.9%) to diatoms. We then calculated a limitation index from N:Fe ratios (log_10_(N/Fe)) which has emerged as an effective empirical means to determine N limitation, Fe limitation or N–Fe co-limitation^57^. Values close to zero indicate N–Fe co-limitation, whereas log_10_(N/Fe) > 0 indicates Fe limitation and log_10_(N/Fe) < 0 indicates N limitation of the phytoplankton community. In contrast to results for all eukaryotic microbial rhodopsins, Spearman’s rank correlation analysis between diatom rhodopsin transcript abundances and environmental factors showed a negative correlation with Fe (*r*_(82)_ = −0.27, *P* = 0.025), and positive correlations with Si (*r*_(82)_ = 0.23, *P* = 0.047) and the log_10_(N/Fe) limitation index (*r*_(82)_ = 0.43, *P* = 2 × 10^−4^) (Fig. 6d; no significant correlation was found with temperature (*P* = 0.08)). Taken together, our results suggest that diatom rhodopsins play a particularly important physiological role in shaping their adjustments to iron stress in the Southern Ocean, which is chronically depleted in dissolved Fe^35,58^.

## Discussion

This study provides evidence that light-driven xanthorhodopsins enhance the growth and biomass of diatom species in iron-limited surface oceans. As the latter represent ~35% of the global ocean surface, our results contribute to the understanding of drivers of marine ecosystem productivity. Thus, alongside previously identified strategies of modifications to the relative abundance of photosystem I and II^39–41^ and replacement of iron-containing ferredoxin and cytochrome *c*_6_ with metal-free flavodoxin and copper-containing plastocyanin, respectively^37,38^, xanthorhodopsins appear to provide another evolutionary strategy for accommodating growth under iron-limiting conditions. In contrast to a previous 'omic study on a variety of eukaryotic phytoplankton^59^, we additionally find a statistically supported correlation of metatranscriptome-derived diatom xanthorhodopsin abundances with dissolved Fe concentrations. This discordance may be due to sampling of different geographic regions, or methods for identifying diatom rhodopsins^60^. Moreover, while previous studies on microbial rhodopsins from diatoms and dinoflagellates such as *Pseudo-nitzschia granii*^61^ and *Polarella glacialis*^62^, respectively, identified proton-pumping activity, we provide experimental evidence for their biological role and importance for adaptation to environmental conditions.

Our studies are particularly relevant for the Southern Ocean, which is both the largest iron-limited aquatic ecosystem and among the most productive, supporting the largest populations of consumers such as krill, fish, penguins and whales. The genome of *F. cylindrus*, as a keystone primary producer in the Southern Ocean, has revealed that genomic complexity, including the divergence of alleles and paralogues, probably contributes to success in this extreme environment^33^. Our results exemplify how the *Fc*R1 xanthorhodopsin generally provides a fitness advantage for primary producers in iron-limited waters. They also illustrate how sequence divergence of two gene copy variants relates to coping with specific environmental conditions, although the biological role of *Fc*R2 remains elusive. The two gene copy variants rendered different expression patterns in our experiments, and their presumed sub- or neo-functionalization appears to be related to a single non-synonymous nucleotide polymorphism that introduces a stop codon at the *Fc*R2 N terminus, resulting in an N-terminal truncation of 30 amino acids. Otherwise, the amino acid sequences of both variants are identical, including the transmembrane regions and conserved residues, and both can function as proton pumps.

Although native subcellular targeting signal sequences have been predicted for algal rhodopsins^63^, our study provides experimental evidence for plastid localization as evidenced through subcellular targeting analysis. Nevertheless, additional work will be needed to determine the precise localization inside the complex plastids of diatoms. Because this is essential information for determining the mechanism by which it enhances growth, we developed a conceptual model to discuss the potential mechanisms that lead to the observed growth enhancement under iron limitation (Fig. 6e). Two scenarios appear possible based on its biophysical characterization in *Xenopus* oocytes: (1) localization in the thylakoid membrane and (2) localization in one of the outer plastid membranes. The essential all-*trans*-retinal would be available for either localization because it can be synthesized from β-carotene by a putative plastid-targeted β-carotene dioxygenase (Extended Data Fig. 1). For scenario (1), *Fc*R1 would presumably be oriented so that the thylakoid lumen would be akin to the extracellular side in a prokaryotic cell. Under green light, *Fc*R1 would then transport protons from the plastid stroma into the thylakoid lumen independently of cellular iron availability to generate a trans-thylakoidal proton gradient driving chloroplast ATP synthases. Hence, *Fc*R1 and homologous proton-pumping rhodopsins would compensate for the iron limitation impact of a reduced proton gradient in the photosynthetic electron transport chain, which is strongly dependent on iron availability (for example, 24 Fe atoms per individual photosynthetic electron transport chain) (Fig. 6e). Under scenario (2), if *Fc*R1 is localized in one of the four outer plastid membranes oriented towards the cytosol, it would probably contribute to generating a proton motive force facilitating the uptake of dissolved iron and therefore supporting photosynthetic electron transport under iron scarcity. Both scenarios contribute to increased production of ATP under iron-limited growth if iron uptake is suboptimal, which depends on the range of iron transporters, their association with prevalent forms of available iron and the cellular iron demand.

In conclusion, we have characterized two gene copy variants (*Fc*R1/2) of xanthorhodopsin from the cold-adapted diatom *F. cylindrus*. We find that the structural evolution of *Fc*R1 has enhanced its efficiency under lower temperatures on the basis of temperature-dependent changes in the photocurrents, a finding that aligns well with biophysical measurements conducted with a proteorhodopsin HSG119 from an Antarctic marine bacterium^64^. The two variants exhibit differences, with *Fc*R1 being expressed only under iron-limited conditions and experimentally demonstrated green-light-activated proton transport. Collectively, these insights suggest a benefit of rhodopsin-enhanced phototrophy for diatoms, especially in iron-limited regions supporting recent findings^43^. Future challenges include understanding the functional role of the variant *Fc*R2, which is more ubiquitously expressed, and the intricacies of rhodopsin phototrophy relative to chlorophyll-based photosynthesis during iron limitation. Recognizing the inherent intertwining with ocean warming and our findings on cold optimization of the xanthorhodopsins characterized herein, we postulate that the importance of diatom rhodopsins in iron-limited oceans may increase as oligotrophic areas expand and iron availability is modified in response to ongoing global change^65,66^, external inputs^67^ and ocean acidification^68^.

## Methods

### Protein sequence and prediction analysis

The amino acid sequence of *Fc*R was deduced from the *F. cylindrus* genome sequence^33^. Protein alignments with characterized microbial rhodopsins were performed using the MUSCLE algorithm implemented in Geneious (v.5.6)^69^. Predictions of subcellular location were performed with SignalP v.4.0 and TargetP (v.1.1)^70^. Putative transmembrane domains and secondary (2D) structure predictions were performed using CCTOP^71^ and visualized with Protter (v.1.0)^72^ with manual modifications. A predicted (3D) structure of *Fc*R1 was generated using ColabFold^73^. The Protein Data Bank (PDB, http://rcsb.org)^74^ was scanned for homologous structures using a Dali search^47^. The experimental structure of PDB entry 4HYJ^42^, a top five search hit, was manually chosen on the basis of highest quality according to PDB indicators and used for structural alignment with *Fc*R1 using ChimeraX^75^ to position the retinal in the predicted *Fc*R1 structure. The final *Fc*R1 model was generated by removing the structure of PDB ID 4HYJ and visualized using ChimeraX.

### Phylogenetic analysis and placement of rhodopsin metatranscripts

Rhodopsin sequences focusing on eukaryotic xanthorhodopsin protein sequences were collected from the literature^2,11,76^ and supplemented with manually curated sequences from BlastP search results of the *Fc*R1 protein sequence against NCBI databases^77^, a custom database (A. Rozenberg and O. Beja, unpublished), as well as similar sequences at an amino acid sequence identity threshold of ≥50% based on their membership in the UniRef50 cluster built using *Fc*R1 as seed sequence (UniRef50_A0A7S2RIV1)^78^. The selected set of proteins was further supplemented with a manually curated set of outgroup sequences including characterized members of the proteorhodopsin subfamily.

To assess the physiological significance of proton-pumping xanthorhodopsins, we searched predicted proteins from 82 upper-ocean eukaryotic metatranscriptome sampling sites^53,54^ that had been functionally annotated using the Pfam database^79^ by the US Department of Energy (DOE) Joint Genome Institute (JGI) for sequences annotated as bacteriorhodopsin-like proteins (Pfam: PF01036).

To only select the rhodopsin sequences attributed to diatoms in the proteins predicted from metatranscriptomes, we used a phylogenetic placement approach as a filter to retain sequences for downstream analyses: the massively parallel Evolutionary Placement Algorithm (EPA-ng). In the rhodopsin reference dataset, one sequence labelled as a diatom (from the MMETSP0851 transcriptome) appeared to be mislabelled as a probable contamination from a dinoflagellate. Sequences were aligned using MAFFT with default parameters^80^ and positions with 25% or more gaps were masked. The maximum-likelihood (ML) reference reconstruction was built using RAxML (v.8.2)^81^ under the LG + G4 model (selected using IQ-TREE^82^ with the corrected AIC). A total of 58,316 metatranscriptomic sequences assigned as proteorhodopsin in amino acid space were then aligned against the unmasked reference tree sequences using the –addfragment option in MAFFT^83^. Then, these aligned metatranscriptomic sequences were phylogenetically placed onto the reference ML reconstruction using EPA-ng (v.0.3.6)^84^, which employs a RAxML evolutionary placement algorithm under the LG model. Only the sequences that were placed onto the diatom branches of the reference tree were kept for downstream analyses.

### Phytoplankton strains, media and growth conditions

The phytoplankton strains of *F. cylindrus* (strain CCMP 1102) and *T. pseudonana* (strain CCMP 1335) used were obtained from the Provasoli-Guillard National Centre for Marine Algae and Microbiota (NCMA (formerly CCMP), https://ncma.bigelow.org/). *P. tricornutum* (strain UTEX 646) was obtained from the University of Texas at Austin (UTEX) Culture Collection of Algae^85^.

*F. cylindrus* cell culture experiments were performed as described previously^31,33,86^. In addition to the previously described experimental treatments including (1) optimal growth (4°C, nutrient replete, 24 h light at 35 μmol photons m^−2^ s^−1^), (2) freezing temperatures (−3°C, nutrient replete, 24 h light at 35 μmol photons m^−2^ s^−1^), (3) elevated temperatures (11°C, nutrient replete, 24 h light at 35 μmol photons m^−2^ s^−1^), (4) elevated carbon dioxide (4°C, 1,000 ppm CO_2_, 24 h light at 35 μmol photons m^−2^ s^−1^), (5) iron starvation (4°C, −Fe, 24 h light at 35 μmol photons m^−2^ s^−1^) and (6) prolonged darkness (4°C, nutrient replete, 7 d darkness), we subjected *F. cylindrus* to (7) growth at half-saturation with silicate (4°C, 0.3 μM silicate, 24 h light at 35 μmol photons m^−2^ s^−1^) as well as (8) red (4°C, nutrient replete, 24 h light at 35 μmol photons m^−2^ s^−1^, 550–700 nm filter) and (9) blue light illumination (4°C, nutrient replete, 24 h light at 35 μmol photons m^−2^ s^−1^, 480–540 nm filter). Experimental *F. cylindrus* cultures were sampled for RNA preparations during mid-exponential phase (~500,000 cells per ml). Different light environments were created by wrapping culture vessels in commercial colour filters (Lagoon Blue 172 and Sunset Red 025, LEE Filters) or illuminating cultures with red and green LED lamps (Crompton). Light spectra were determined using a spectroradiometer (SR9910, Macam Photometrics). For batch culturing of *F. cylindrus* under half saturation with silicate, the nutrient was regularly resupplied to a final concentration of 0.3 μmol l^−1^ during the experiment. Before the batch culturing, the half-saturation constant *K*_m_ of *F. cylindrus* for silicate was determined by growing cells over a concentration range of 0.01–100 μmol l^−1^ silicate (Supplementary Fig. 1).

*T. pseudonana* cell culture experiments were performed analogous to that of *F. cylindrus* using iron-free Aquil* medium^87^, adding trace metals stock solution without iron that was prepared separately (1 mM FeCl_3_ × 6H_2_O, 100 μM EDTA) and added at final iron concentrations. The medium was supplemented with all-*trans*-retinal (Sigma-Aldrich) at a final concentration of 3 μmol l^−1^ from a 10 mM retinal stock dissolved in 100% dimethyl sulfoxide. *T. pseudonana* cultures were grown in a 20°C incubator under 24 h light at 38 µmol photons m^−2^ s^−1^ white light. Cells were maintained and grown in the iron-replete Aquil* media (1,000 nmol l^−1^ iron). Before iron-limitation experiments, the *T. pseudonana* cultures were transferred into iron-free Aquil* media and incubated for 5 d. The iron-starved cultures were then transferred into either iron replete (1,000 nmol l^−1^ iron, 3 μmol l^−1^ retinal) or iron-limited Aquil* media (150 nmol l^−1^ iron, 3 μmol l^−1^ retinal) at an initial concentration of ~8 × 10^5^ cells per ml. Five replicates each were used for control and treatment groups.

Cell abundance over time was estimated by a Multisizer Coulter counter (Beckman Coulter). Specific growth rates per day (*μ*) were calculated from the linear regression of the natural log of cell abundances (cells per ml) over time during the exponential growth phase or (when using only two sampling points) according to the following equation:

1 $$\mu ({d}^{-1})=\frac{\mathrm{ln}\,{N}_{t2}-\mathrm{ln}\,{N}_{t1}}{\Delta t},$$

where *N*_*t*1_ denotes the cell abundance at timepoint *t*_1_, *N*_*t*2_ the cell abundance at timepoint *t*_2_ and Δ*t* the time difference between the two timepoints^88^.

The maximum quantum yield of photosystem II (*F*_v_/*F*_m_) was measured using pulse-amplitude-modulated (PAM) fluorometry, using a Phyto-PAM fluorometer equipped with a Phyto-ED measuring head (Walz). The in vivo quantum yields were determined in each culture and calculated using PhytoWin software (v.2.00a, Walz) from fluorescence readings of dark-acclimated samples.

### RNA extraction from *F. cylindrus*

Total RNA was extracted using guanidinium thiocyanate-phenol-chloroform extraction^89^ and TRI reagent (Sigma-Aldrich), followed by DNase I (Qiagen) treatment (1 h, 37°C) and purification using RNeasy MiniElute cleanup kits (Qiagen) according to manufacturer instructions. The purity of RNA was checked on a NanoDrop spectrophotometer (Thermo Fisher) and integrity using 2% denaturing formaldehyde gels or an Agilent 2100 Bioanalyzer (Agilent). RNA concentrations were determined in duplicate readings using a NanoDrop spectrophotometer.

### RT–qPCR

RT–qPCR to determine *F. cylindrus* rhodopsin gene expression levels was performed using a two-tube RT–qPCR protocol^90^.

First-strand cDNA synthesis was performed using Superscript II reverse transcriptase (Invitrogen) utilizing Anchored Oligo(dT)_20_ primer (Invitrogen) or Oligo(dT)_20_ primer (Invitrogen). Reverse transcription of 500 ng of total RNA was carried out in 50 μl reactions at 42°C for 50 min, followed by inactivation at 70°C for 15 min. As a control for DNA contamination, RNA was pooled from each biological replicate and first-strand synthesis reaction mix was added, omitting reverse transcriptase.

Oligonucleotides (Supplementary Table 1) were designed towards the 3’ end of the gene of interest using the web-based RealTimeDesign software (Biosearch Technologies, http://www.biosearchtech.com/realtimedesign) aiming for an amplicon length of 80–150 bp (optimum 115 bp), a GC content of amplicon and primer of 30–80%, a primer length of 18–30 bp and a primer melting temperature *T*_M_ of 63–68°C. BLAST searches of the primer sequences against the *F. cylindrus* genome sequence (https://mycocosm.jgi.doe.gov/Fracy1/Fracy1.home.html) were performed and, if necessary, primer sequences were modified manually to ensure maximum specificity. Oligonucleotides were assessed for *T*_M_, hairpins and primer dimers using the web-based tool OligoAnalyzer 3.1 (Integrated DNA Technologies, http://eu.idtdna.com/analyzer/Applications/OligoAnalyzer), parameterized with oligo concentrations of 0.4 μM, Na^+^ of 50 mM, Mg^++^ of 5.5 mM and deoxynucleotide triphosphates of 0.5 mM. Primers were synthesized by Eurofins Genomics.

For qPCR reactions and second-strand amplification, 5 μl of a 10-fold diluted reverse transcriptase reaction mix was supplemented with 20 μl 2× SensiMix SYBR Green NoROX master mix (Bioline). Forward and reverse primers were added at a concentration of 200 nM. Amplifications were performed in white 96-well plates on a CFX96 real-time system (Bio-Rad) using the following conditions: initial denaturation at 95°C for 10 min, followed by 40 amplification and quantification cycles of 15 s at 95°C, 15 s at 59°C, 10 s at 72°C. Finally, a melting curve analysis (65°C to 95°C, increments of 0.5°C, dwelling time 5 s) was carried out to check for primer dimers and non-specific amplification. For each primer pair, the reliability of qPCR was demonstrated by five-to-six-point standard curves made by amplification from 1:10 serial dilutions of reverse transcription reactions. Standards for absolute RT–qPCR gene expression analysis were generated as follows: target sequences were amplified using conventional PCR from cDNA or plasmid templates, separated by agarose gel electrophoresis and purified (illustra GFX PCR DNA and Gel Band Purification kit, GE Healthcare). The concentration of agarose gel-purified target sequences was determined in duplicate readings using a NanoDrop spectrophotometer (Thermo Fisher) and diluted to 1:10,000. Subsequently, six-point standard curves were determined for specific target sequences by qPCR amplification from 1:10 serial dilutions of the prepared 10,000× dilution. The absolute amount of cDNA in the samples was calculated on the basis of the equation obtained for logarithmic regression lines for standard curves.

### Variant-specific qPCR

Variant-specific qPCR to discriminate between expressions of nearly identical gene copies in *F. cylindrus* was performed^91^. Briefly, the specificity of the PCR amplification was conferred by placing the 3’-end of a forward or the reverse variant-specific primer directly over a single nucleotide polymorphism but matching one or the other variant. Then variant-specific qPCR was performed in two separate reactions using a common primer and either an *Fc*R1-specific primer or an *Fc*R2-specific primer. Although in theory, only completely matching primers should be extended and only the matching variant sequence should get amplified, there would be amplification of the mismatched variant but with lesser efficiency^91^. The more frequent variant would reach the cycle threshold (*C*_t_) at an earlier qPCR amplification cycle (that is, having a smaller *C*_t_) and the difference in *C*_t_ values between the two separate qPCR reactions, the Δ*C*_t_, provides a measure of the variant frequency. The allele frequency was calculated^91^ using the following equation:

2 $${\rm{frequency}\,{of}\,{{variant}}}_{1}=\frac{1}{{2}^{\Delta {C\rm{t}}}+1},$$

where $\Delta {C}_{\rm{t}}=({C}_{\rm{t}}\,{\rm{of}\,{{variant}}}_{1}\,{\rm{specific}\,{qPCR}})-({C}_{\rm{t}}\,{\rm{of}\,{{variant}}_{2}\,{specific}\,{qPCR}})$ describes the difference in *C*_t_ values between the two qPCR reactions.

Generally, variant-specific primers were designed as described above. However, in addition to their specific design to match only one of the variant sequences at its 3’-terminal nucleotide, additional nucleotide mismatches located three bases from the 3’-end of the variant-specific primer were incorporated to improve amplification specificity as performed previously^92,93^. The variant-specific primers used in this study are described in Supplementary Table 2.

To validate our variant-specific qPCR, a prepared standard mix containing known ratios of plasmid DNAs encoding cloned full-length sequences of either *Fc*R variant was used. Therefore, pPha-T1 plasmids (pPha-T1 *Fc*R1::GFP and pPha-T1 *Fc*R2::GFP) were linearized (KpnI restriction digest, 37 °C, 3 h) to avoid strong biases by circular (supercoiled) plasmid standards^94^. DNA concentrations of purified linearized plasmid (illustra GFX PCR DNA and Gel Band Purification kit, GE Healthcare) were determined in three technical replicates using a NanoDrop spectrophotometer (Thermo Fisher). Subsequently, 10 μl of purified linearized plasmids were concentrated until dry using a centrifugal evaporator (miVac DNA concentrator, Genevac), and 10 nM standard solutions of each plasmid were set up with molecular-grade water. Subsequently, 0.001 nM (1 pM) plasmid standards were made using 1:10 serial dilutions of 10 nM standards. Standard mixtures of 100 μl were made up from both 1 pM plasmid standards to contain *Fc*R variants with known copy frequencies of 0.05, 0.1, 0.2, 0.3, 0.4, 0.5, 0.6, 0.7, 0.8, 0.9 and 0.95, which were used as templates for qPCR as described above but with an annealing temperature of 60°C.

### Production of *F. cylindrus* rhodopsin antiserum

Antiserum against *Fc*R1/2 was produced using synthetic antigenic peptides (Standard immunization programme, Eurogentec). Polyclonal antibodies to *Fc*R1/2 were raised against the peptides GELAKTGSPFNDAYR (extracellular loop between helix II and helix III, residues 104–118) and KSLALKNGNEETPLL (C terminus, residues 275–289) and used to immunize two different rabbits each. A cysteine residue was coupled to the C and N termini of the antigenic peptides for coupling to m-maleimidobenzoic acid *N*-hydroxysuccinimide ester conjugated to keyhole limpet haemocyanin carrier protein. IgGs from the rabbit sera were affinity purified after coupling to Toyopearl AF-amino resin, washing with phosphate-buffered saline (PBS; 137 mM NaCl, 2.7 mM KCl, 10 mM Na_2_HPO_4_, 1.8 mM KH_2_PO_4_) and elution with 100 mM glycine pH 2.5. Immunoreactivity against the antigen was assessed by subsequent western blotting, obtaining best results with the unpurified antiserum.

### Protein extraction and western blot analysis

Membrane protein extractions were prepared from 100 ml of mid-exponential *F. cylindrus* cell cultures (~5 × 10^5^–1 × 10^6^ cells per ml) collected by centrifugation (3,000 *g*, 10 min at 4°C) using the Mem-PER Plus membrane protein extraction kit (Thermo Fisher) according to manufacturer instructions. The total protein concentrations were determined using a bicinchoninic acid (BCA) assay (Pierce BCA protein assay kit, Thermo Fisher). A protein sample of 30–50 μg was mixed with 6× Laemmli SDS–PAGE sample loading buffer (375 mM Tris-HCl pH 6.8, 10% (w/v) SDS, 60% glycerol, 0.06% bromophenol blue) supplemented with reducing agent β-mercaptoethanol in a total volume of 40–50 μl and directly loaded on 12% SDS–PAGE gels without boiling to avoid membrane protein oligomerization. In addition, 10 μl of a molecular weight marker (Precision Plus Protein Dual Color Standards, Bio-Rad) were loaded and proteins were transferred to nitrocellulose or PVDF membranes using a Criterion Blotter (Bio-Rad). Equal sample loading was checked by total protein staining, as recommended^95^, with Ponceau S stain (0.5% (w/v) Ponceau S, 1% acetic acid) for 20 min at room temperature and rinsing with milli-Q water. The membrane was blocked with 5% milk in PBS containing 0.1% (w/v) Tween 20 (PBST) for at least 1 h at room temperature or overnight. The *Fc*R1/2 protein was detected by blotting the membrane with a 1:1,000 dilution of rabbit polyclonal anti-*Fc*R1/2 antiserum with 5% milk in PBST for 1 h at room temperature, then washed three times with PBST for 10 min, followed by blotting with a 1:10,000 dilution of secondary horseradish peroxidase-conjugated anti-rabbit IgG. Three additional washes with PBST of 10 min were done before visualizing immune complexes with the Amersham enhanced chemiluminescence kit (Thermo Fisher) and a LAS-3000 imager (Fujifilm).

### PCR amplification of *F. cylindrus* rhodopsin variants

A full-length product of the *Fc*R1 variant was amplified from cDNA, which was synthesized from RNA of iron-limited *F. cylindrus*, using touchdown PCR amplification with the forward primer 5’-CCTTTTACCGTACAATGCGAGAG-3’ and reverse primer 5’-CAAAATCTGACACTAGGCCCTACC-3’ and successive annealing temperatures of 72°C (5 cycles), 70°C (5 cycles) and 62°C (30 cycles). Touchdown PCR reactions were performed with Phusion DNA polymerase (Finnzymes) according to manufacturer recommendations but using a 1:1 mixture of HF and GC buffer as well as addition of 3% dimethyl sulfoxid and 1 μg μl^−1^ BSA.

The full-length *Fc*R2 variant was amplified from ~100 ng cDNA template with proofreading DNA polymerase (Pfu, Fermentas), using the forward primer 5’-ATGATCAGC GGAACTCAATTCAC-3’ and reverse primer 5’-AAGGAGAGGAGTTTCTTCGTTTC-3’. A 50 μl reaction contained 0.4 pM of each primer, 0.2 pM of each deoxynucleotide triphosphate, 1× Pfu buffer and 10 mM MgSO4. The amplification profile was as follows: 4 min initial denaturation at 95°C, followed by 35 cycles of 95°C for 45 s, 55°C for 45 s, 72°C for 90 s extension and final extension at 72°C for 5 min.

After purification of agarose gel fragments, gene-specific primers containing restriction sites were used for directed cloning and subcloning of *Fc*R gene constructs into vectors for heterologous expression in diatoms and *X. laevis* oocytes (Supplementary Figs. 2–5). Expression vectors were transformed and maintained in *E. coli*. Plasmids from *E. coli* were isolated by the alkaline extraction method^96^ using commercial plasmid prep kits. The orientation and accuracy of expression constructs was verified by small-scale capillary sequencing.

### Plasmids for in vitro RNA synthesis

The DNA fragments of *Fc*R1 and *Fc*R2 were amplified by PCR with primers carrying the corresponding restriction sites and digested before being inserted into the oocyte expression vectors pGEMHE (Supplementary Fig. 2) carrying different tags. The sequences were confirmed by sequencing. NheI-linearized plasmid DNA was used for the in vitro generation of complementary RNA (cRNA) following the protocol of the AmpliCap-MaxT7 High Yield Message Maker kit (Epicentre Biotechnologies) for oocyte injection.

### TEVC experiments

Conserved residues of ion-transporting rhodopsins can also be present in sensory rhodopsins^21,97,98^. Hence, it is necessary to provide evidence for their proton-pumping activity by experimental characterization using heterologous expression systems such as *Xenopus* oocytes^99–101^. To test whether both *Fc*R gene copies function as proton pumps, we expressed them individually in *Xenopus* oocytes for electrophysiological characterization using TEVC measurements. Indeed, photocurrents were observed in some oocytes expressing *Fc*R1 wild type upon illumination with a 532 nm diode-pumped solid state (DPSS) laser. Despite the observed photocurrents of *Fc*R1, targeting the protein to the plasma membrane and its expression in *Xenopus* oocytes had to be optimized by applying protein engineering strategies including membrane trafficking signal sequences. We found that N-terminal fusion of a rat gastric H^+^/K^+^-ATPase β-subunit fragment (amino acids 1–105, ref. ^102^) to an N-terminally truncated *Fc*R1 enhanced targeting to the oocyte plasma membrane. We also found that C-terminal eYFP fusions and epitope tagging either abolished or strongly reduced *Fc*R1 photocurrents in oocytes, which we solved by inserting the Golgi apparatus trafficking signal from the Kir2.1 K^+^ channel between the truncated *Fc*R1 and eYFP sequence. Altogether, we achieved the best results by a combination of both, fusing the H^+^/K^+^-ATPase β-subunit fragment together with amino acids 1–10 from *Coccomyxa subellipsoidea* rhodopsin (*Cs*R) to the N terminus of the truncated *Fc*R1 and fusing the Golgi apparatus trafficking (T) signal from the Kir2.1 K^+^ channel, an eYFP (Y) tag and the Kir2.1 endoplasmic reticulum export (E) signal to its C terminus.

For expression of all constructs, 30 ng of cRNA were injected into *Xenopus* oocytes. cRNA-injected oocytes were incubated in ND96 solution (in mM, 96 NaCl, 5 KCl, 1 MgCl_2_, 1 CaCl_2_, 5 HEPES, pH 7.4) containing 1 µM all-*trans*-retinal at 16°C. Photocurrents were measured 2 or 3 d after injection with the light source from a 530 nm LED (Thorlabs) or a 532 nm DPSS laser (Changchun New Industries Optoelectronics). The light intensities were measured with a PLUS 2 power and energy metre (LaserPoint Srl). Electrophysiological measurements were performed at room temperature (20–23°C) (otherwise indicated in the legend) with a TEVC amplifier (TURBO TEC-05, npi electronic). The bath solutions are indicated in figure legends. Electrode capillaries (*Ф* = 1.599 mm, wall thickness 0.178 mm, Hilgenberg) were filled with 3 M KCl, with tip openings of 0.2–1 MΩ. A USB-6221 DAQ device (National Instruments) and WinWCP (v.5.5.3, Strathclyde University, United Kingdom) were used for data acquisition.

### Action spectrum

Light of different wavelengths was obtained from a PhotoFluor II light source (89 North) together with narrow bandwidth interference filters (Edmund Optic) of different wavelengths. The light intensities at different wavelengths were measured with a Laser Check photometer (Coherent Technologies).

### Oocyte imaging

The expression levels of rhodopsin proteins in oocytes were estimated from the C-terminally fused YFP 2 d after cRNA injection. The images of *Xenopus* oocytes were obtained using a confocal laser scanning microscope (LSM 5 Pascal, Zeiss) equipped with a Plan-Neofluar ×10/0.5 objective (Zeiss). The fluorescence images were processed using the LSM 5 image browser and the pictures were exported for insertion into figures.

### Subcellular localization of *Fc*R1 in diatoms

The *Fragilariopsis* rhodopsin gene was cloned into the *P. tricornutum* expression vector pPha-T1 (ref. ^103^) for analysis of subcellular targeting using green fluorescent protein (GFP) labelling^104^. PCR-amplified products *Fc*R1 were purified (illustra GFX PCR DNA and Gel Band Purification kit, GE Healthcare) from 1.2% TAE agarose gels (40 mM Tris acetate, 1 mM EDTA, 0.5 μg ml^−1^ ethidium bromide) and ligated into the *P. tricornutum* transformation vector StuI-GFP pPha-T1 (refs.^103,105^) (Supplementary Figs. 3 and 4) using blunt-ended non-directional ligation with StuI (Eco147I) restriction enzyme. Restriction digest with StuI and ligation was performed in a single tube. Therefore, a 20 μl reaction mix (1 μl vector, 5 μl insert, 1 μl StuI restriction enzyme, 2 μl PEG 4000, 2 μl ATP/DTT (10 mM/100 mM), 2 μl restriction enzyme buffer, 1 μl T4 ligase; adjusted to 20 μl with molecular-grade water) was incubated overnight at room temperature until the reaction was inactivated by heating to 65°C for 20 min. After cooling down to room temperature, empty vector molecules were digested by adding 1 μl StuI restriction enzyme to the inactivated reaction mix and incubating for 1.5 h at 37°C. The digest of empty StuI-GFP pPha-T1 vector molecules was inactivated by heating to 65°C for 20 min. After cooling down to room temperature, 7 μl of the ligation reaction mix was transformed into CaCl_2_-competent *E. coli* DH5α cells and selected on ampicillin (100 μg ml^−1^). Colony PCR using a combination of insert and vector primers was used to screen for plasmids with correct orientation of insert. In addition, *Fc*R1 was amplified and cloned into the vector pTpFCP-GFP/fcpNat (Supplementary Fig. 5) for constitutive expression in *T. pseudonana*^51,106^ with primers carrying the corresponding restriction sites. Plasmid and primer sequences were managed with Geneious v.2023.0 (Biomatters, https://www.geneious.com).

Nuclear transformation of *P. tricornutum* and *T. pseudonana* was performed using a Biolistic PDS-1000/He particle delivery system (Bio-Rad) fitted with 1,350 psi rupture discs as described previously^51,104,107^. For the selection and cultivation of *P. tricornutum* transformants, 75 μg ml^−1^ Zeocin (InvivoGen) was added to the solid 1.2% agar medium. To detect green fluorescence signals from GFP-transformed diatom cell lines, flow cytometry (FACScalibur, BD) with the standard optical filter configuration was used. Therefore, green fluorescence was measured in the FL1 channel using a 515–545 nm emission filter. FL1 histograms were used to identify transformed (peak ~10^3^) and non-transformed (peak ~10^1^) cells. Milli-Q water was used as a sheath fluid and all analyses were performed using a low flow rate (~20 μl min^−1^). Triggered on green fluorescence, 10,000 events were collected. An event rate between 100 and 400 cells per second was used to avoid coincidence and when needed, samples were diluted in 0.2-μm-filtered artificial seawater before analysis. To confirm the presence of GFP and analyse the morphology of the cells, upright wide-field fluorescence microscopy (Axioplan 2 IE imaging microscope equipped with a CCD Axiocam camera, Zeiss) was performed. Chloroplasts were identified by red autofluorescence of chlorophyll *a*/*c* during excitation at 562 ± 20 nm (Alexa568 filter set). The excitation and emission of filters used during microscopic analyses are listed in Supplementary Table 3.

### Generation of *T. pseudonana Fc*R1 knock-in cell line

*T. pseudonana* wild-type cells (5 × 10^7^ cells per ml) were collected from the exponential growth phase using 0.2 μm Isopore polycarbonate membrane filters (MilliporeSigma). Biolistic transformation of the *Fc*R1 construct followed a previously published protocol^107^. Colonies were selected on nourseothricin agar plates (100 μg ml^−1^ nourseothricin/clonNAT) (Werner BioAgents) prepared with half-salinity Aquil* medium.

Analogous to ref. ^108^, RT–qPCR and DNA sequencing of the PCR product was performed to detect and validate successful transformation and insertion of the *Fc*R1 gene in *T. pseudonana*. A cell culture volume of 80 ml was collected by centrifugation at the end of the exponential growth phase. Collected cells were frozen in liquid nitrogen and lysed by adding 1 ml of Trizol pre-heated to 60°C and using a bead beater mill (BioSpec) for 2 min at room temperature, followed by incubation on ice for 1 min. Total RNA was extracted and purified using the Direct-zol RNA kit and RNA Clean and Concentrator kit (Zymo) according to manufacturer instructions. Before RNA cleanup, genomic DNA was removed by DNase treatment with RNase-free DNase (Zymo) at 37°C for 60 min. A total of 0.2 µg of RNA was used for reverse transcription using the SuperScript IV first-strand synthesis system (Thermo Fisher) according to manufacturer instructions. PCR was then performed with primer pair FR271123 F1 (5’-CGTTTGGCCATTCCTTCATATC-3’) and rhodopsin screen R (5’-GAACCGGAGATTCCCATCATAG-3’). A 50 μl RT–qPCR was prepared according to RED*Taq* ReadyMix PCR Reaction Mix manufacturer instructions (Sigma-Aldrich) and performed in a PCR cycler (Bio-Rad) with initial denaturation for 1 min at 95°C, followed by 35 cycles of 30 s at 95°C, annealing for 30 s at 52°C and elongation for 1 min at 72°C, with a final elongation for 5 min at 72°C. The PCR product was confirmed on a 1% agarose gel and analysed by sequencing (Eurofins Genomics). The *Fc*R1 knock-in mutant cell line was maintained in half-salinity Aquil* medium containing 100 μg ml^−1^ nourseothricin.

### Statistics and reproducibility

Statistical analysis was performed in R (v.4.2.1)^109^. For comparison between specific sets of datapoints, we used parametric and non-parametric testing procedures as indicated in the text. OriginPro v.2020 (OriginLab) was used for analysis of electrophysiological data. A multiple regression analysis using a GLM was performed analogous to that in ref. ^110^. Briefly, we first calculated the abundance of environmental transcripts annotated with bacteriorhodopsin-like protein domains (Pfam: PF01036) and normalized these as a percentage of total transcripts mapped in each metatranscriptome sample. A GLM was then fitted using the glm function of the R stats package (v.4.2.1) using percentages of rhodopsin domains as response variable and temperature, salinity, hemisphere and the transformed nutrients (log(phosphate+0.01), log(nitrate+0.01), log(silicate+0.01) and log(iron+0.01)) as covariates. We then performed a backward elimination of factors by AIC using a stepwise algorithm with the step function of the R stats package. Regression summaries were exported using the jtools R package (v.2.2.0)^111^. Data analysis and visualization were performed in R tidyverse^112^ and using ggplot2 (ref.^113^) and ggtree^114^.

### Reporting summary

Further information on research design is available in the Nature Portfolio Reporting Summary linked to this article.

### Supplementary information

Supplementary Information Supplementary Tables 1–3 and Figs. 1–5.

Reporting Summary

### Source data

Source Data Fig. 1 AlphaFold2 structure prediction of *Fc*R1 with retinal in crystallographic information file format.

Source Data Fig. 2 Newick tree file for maximum-likelihood phylogenetic tree.

Source Data Fig. 3 Source data.

Source Data Fig. 4 Statistical source data for Fig. 4a–k.

Source Data Fig. 5 Statistical source data for Fig. 5c–i.

Source Data Fig. 6 Statistical source data for Fig. 6a–d.

Source Data Extended Data Fig. 1 Statistical source data.

Source Data Extended Data Fig. 2 Source data.

Source Data Extended Data Fig. 3 Statistical source data for Extended Data Fig. 3a–e.

Source Data Extended Data Fig. 4 Source data.

## Data Availability

RNA-seq data are available in the ArrayExpress database (www.ebi.ac.uk/arrayexpress) under accession number E-MTAB-5429. Utilized RNA-seq and metatranscriptome datasets released with previous studies are available in the ArrayExpress database under accession number E-MTAB-5024 and at https://genome.jgi.doe.gov/ (10.25585/1488054). Additional data and metadata including the rhodopsin reference alignment used to taxonomically assign translated rhodopsin metatranscripts, protein structure prediction and vector maps are available at 10.5281/zenodo.8322266. All other data and biological materials are available from the corresponding authors upon reasonable request. Source data are provided with this paper.
