# Supplementary material for: Plastid-localized xanthorhodopsin increases diatom biomass and ecosystem productivity in iron-limited surface oceans
Source: Nat Microbiol. 2023 Oct 16;8(11):2050–66. doi: 10.1038/s41564-023-01498-5 (PMC10627834; doi:10.1038/s41564-023-01498-5)
Supplement: Supplementary file 1 — Supplementary Tables 1–3 and Figs. 1–5. [file 41564_2023_1498_MOESM1_ESM.pdf]

nature microbiology

Article https://doi.org/10.1038/s41564-023-01498-5

Plastid-localized xanthorhodopsin increases diatom biomass and ecosystem productivity in iron-limited surface oceans

In the format provided by the authors and unedited

Supplementary Information

Supplementary Tables

Supplementary Table 1 | Primers used to amplify *Fc*R1/2 by RT-qPCR.

Primer	Sequence (5‘ – 3‘)	Amplicon size (bp)
FcR_555F*	GTTACCGTTCCTCTACATTGTCC	111
FcR_665R	GTCCACCATTGAACACCCTTA	

*contains a single G/A mismatch (underlined) to the *Fc*R1 nucleotide sequence

Supplementary Table 2 | Primers used for variant-specific RT-qPCR.

Primer	Sequence (5’ – 3’)	Amplicon size (bp)
FcR1_271123-C2fw:	GGGTGGTCGTTGGGTC**A**AC	88
FcR1_271123-C2re:	GGTGGCGTCATTGAGT**G**CA	
FcR2_274098-C2fw:	AGGTGGTCGTTGGGTC**A**AT	88
FcR2_ 274098-C2re:	AGTGGCATCGTTAAGT**T**CC	

Note. Primer names consist of gene name, JGI protein ID, code for additional mismatch at 3’-end (e.g., C0: no mismatch, C2: mismatch three nucleotides from 3’-end), abbreviation fw: forward primer, and re: reverse primer). Underlined nucleotides indicate the sites of polymorphisms to the other variant. Bold nucleotides indicate additionally introduced nucleotide mismatches three bases from the 3’- terminus to increase primer specificity.

Supplementary Table 3 | Optical filter sets used in fluorescence microscopy.

Filter	Excitation (nm)	Emission (nm)
UV	365 ± 30	445 ± 30
GFP	469 ± 17.5	525 ± 19.5
Alexa568	562 ± 20	624 ± 20

Supplementary Figures

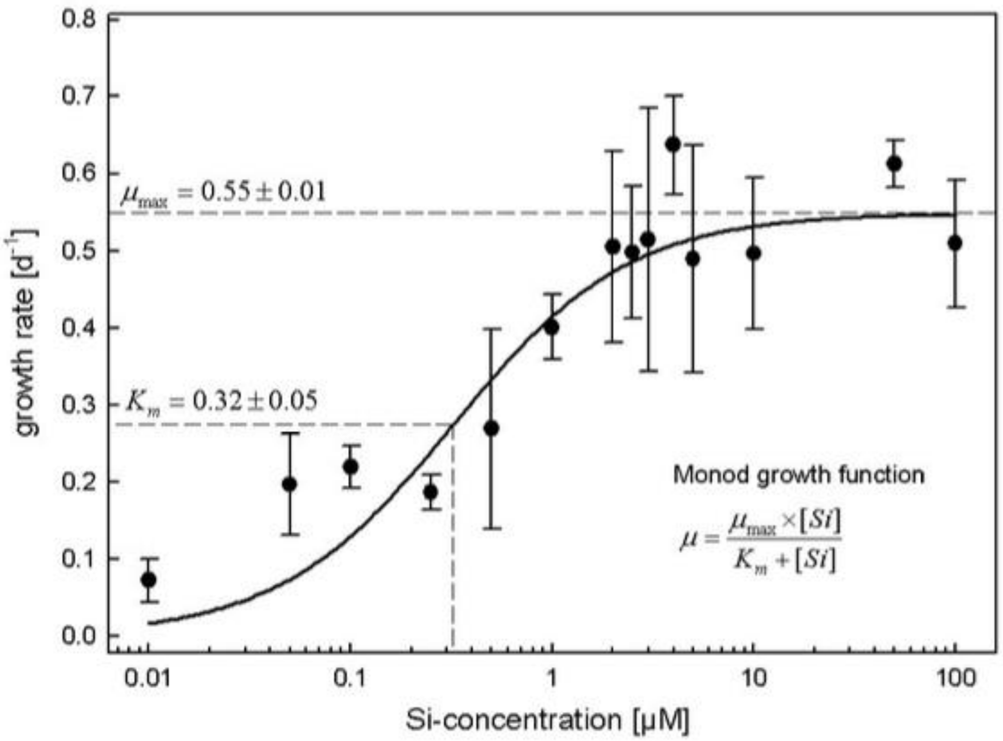

Supplementary Fig. 1 | *Fragilariopsis cylindrus* mean growth rates (d^{-1}) in relation to different silicate [Si] concentrations (log scale). Error bars indicate mean growth rates +/- SD for three ([Si]: 4 µM), four ([Si]: 0.01 µM), six ([Si]: 0.1 µM, 0.25 µM, 2 µM, 50 µM, 100 µM), eight ([Si]: 10 µM), nine ([Si]: 0.05 µM, 2.5 µM, 3 µM), ten ([Si]: 1 µM, 5 µM) and twelve ([Si]: 0.5 µM) independent experiments. Line represents fitting to nonlinear Monod growth function.

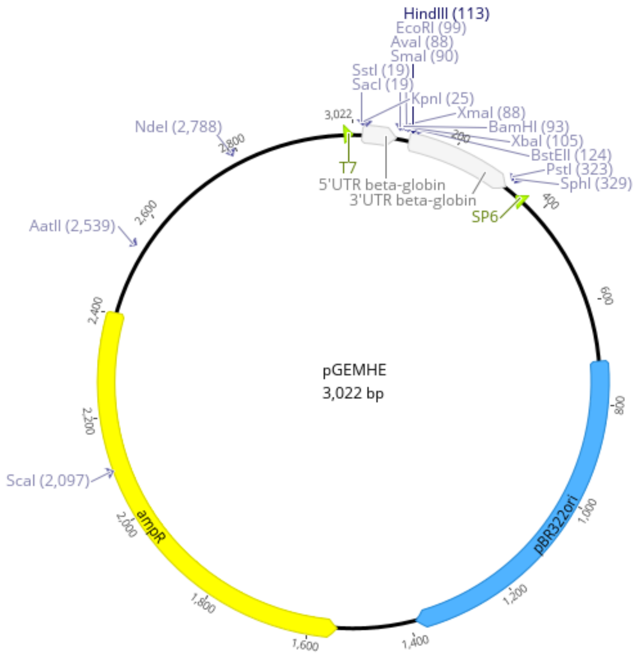

Supplementary Fig. 2 | *Xenopus laevis* expression vector pGEMHE containing 5’ and 3’UTRs from a *Xenopus* β-globin gene (Kreig, P. A., and Melton, D.A. (1984), *Nucl. Acids Res.* 72, 7057-70), which flank a polylinker with restriction enzyme sites (Liman, E. R. et al. (1992), *Neuron* 9(5): 861-71). Plasmid map created with Geneious v2023.0 (Biomatters, Auckland, New Zealand). Available from https://www.geneious.com.

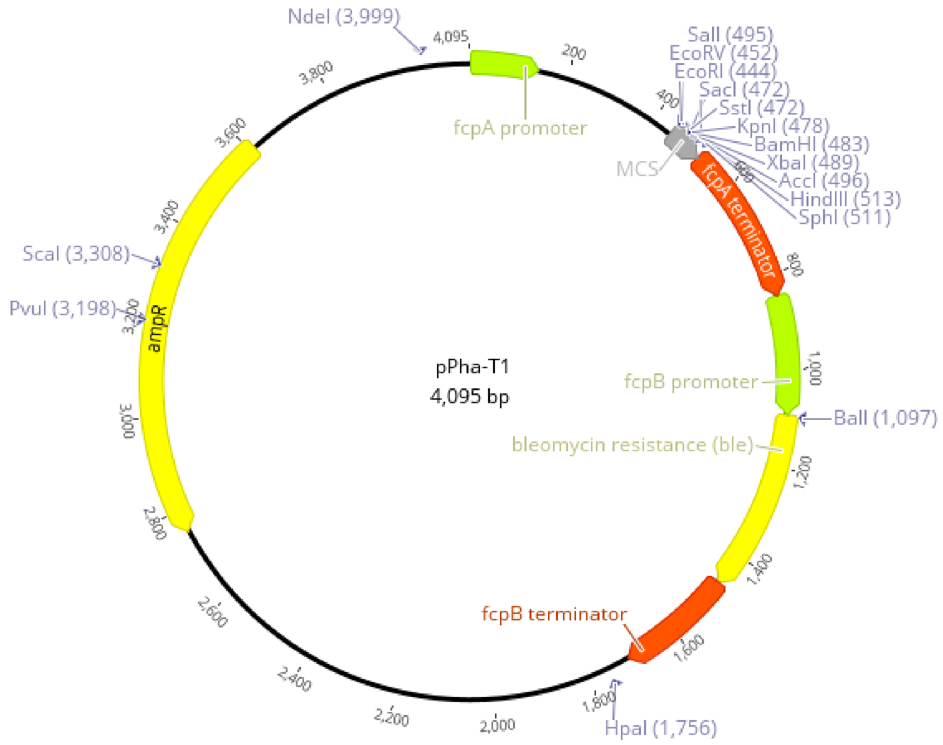

Supplementary Fig. 3 | Phaeodactylum tricornutum transformation vector pPha-T1 (GenBank AF219942; Zaslavskaia et al. (2001), *J. Phycol.* 36(2): 379) containing fucoxanthin chlorophyll-binding protein (fcp) regulatory sequences to drive constitutive expression of bleomycin resistance protein (Ble) conferring zeocin resistance and gene of interest, which is to be cloned into multiple cloning site (MCS). Plasmid map created with Geneious v2023.0 (Biomatters, Auckland, New Zealand). Available from https://www.geneious.com.

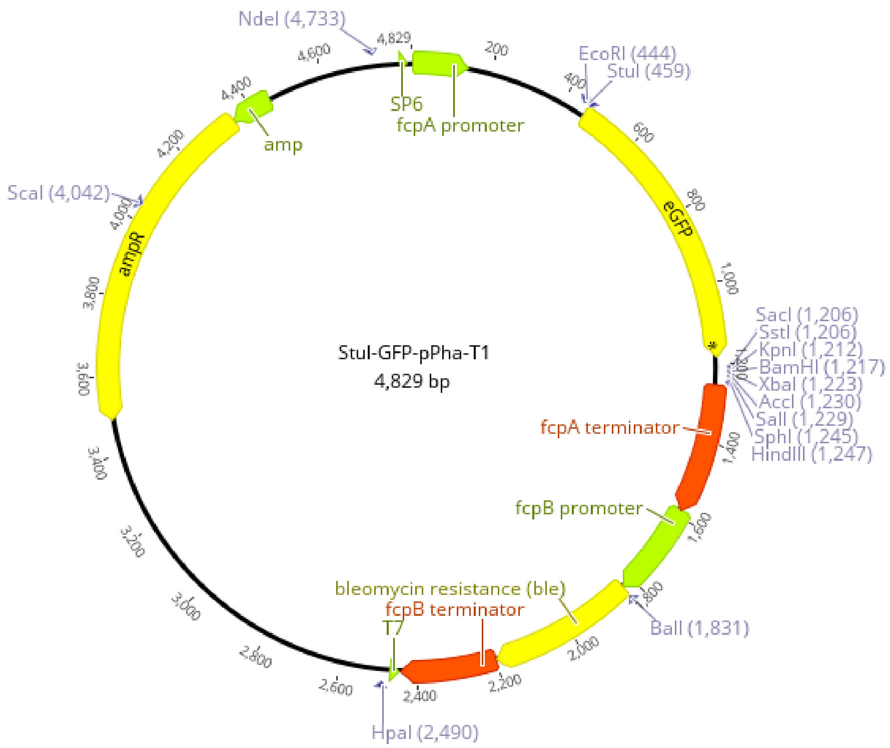

Supplementary Fig. 4 | *Phaeodactylum tricornutum* transformation vector StuI-GFP-pPha-T1 (Gruber et al. (2007), *Plant Mol. Biol.* 64(5): 519) for generation of eGFP fusion proteins. To generate GFP fusion constructs, the sequence of interest is to be cloned into the StuI restriction site at 5' end of eGFP. Vector represents a derivate of the pPha-T1 vector (Zaslavskaia et al. (2001), *J. Phycol.* 36(2): 379). Plasmid map created with Geneious v2023.0 (Biomatters, Auckland, New Zealand). Available from https://www.geneious.com.

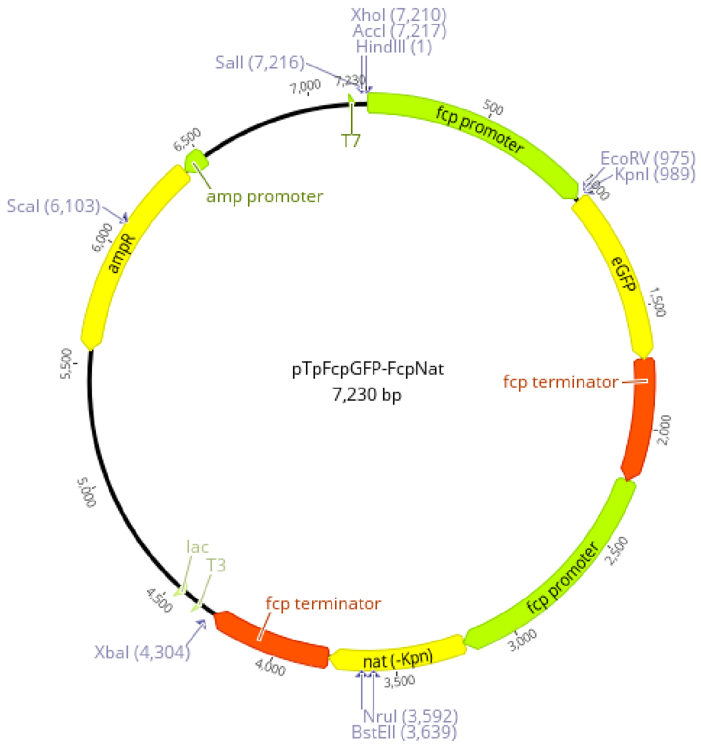

Supplementary Fig. 5 | *Thalassiosira pseudonana* transformation vector pTpFCP-GFP/fcpNat for constitutive expression of eGFP fusion proteins (Poulsen et al. (2006), *J. Phycol.* 42(5):1059; Scheffel et al. (2011), *PNAS* 108(8):3175). Plasmid map created with Geneious v2023.0 (Biomatters, Auckland, New Zealand). Available from https://www.geneious.com.
