# Supplementary material for: Plastid-localized xanthorhodopsin increases diatom biomass and ecosystem productivity in iron-limited surface oceans
Source: Nat Microbiol. 2023 Oct 16;8(11):2050–66. doi: 10.1038/s41564-023-01498-5 (PMC10627834; doi:10.1038/s41564-023-01498-5)
Supplement: Supplementary file 2 — Reporting Summary [file 41564_2023_1498_MOESM2_ESM.pdf]

nature portfolio

Corresponding author(s): Jan Strauss and Thomas Mock

Last updated by author(s): Sep 8, 2023

Reporting Summary

Nature Portfolio wishes to improve the reproducibility of the work that we publish. This form provides structure for consistency and transparency in reporting. For further information on Nature Portfolio policies, see our Editorial Policies and the Editorial Policy Checklist.

Statistics

For all statistical analyses, confirm that the following items are present in the figure legend, table legend, main text, or Methods section.

n/a	Confirmed	
☐	☒	The exact sample size (*n*) for each experimental group/condition, given as a discrete number and unit of measurement
☐	☒	A statement on whether measurements were taken from distinct samples or whether the same sample was measured repeatedly
☐	☒	The statistical test(s) used AND whether they are one- or two-sided<br>*Only common tests should be described solely by name; describe more complex techniques in the Methods section.*
☐	☒	A description of all covariates tested
☐	☒	A description of any assumptions or corrections, such as tests of normality and adjustment for multiple comparisons
☐	☒	A full description of the statistical parameters including central tendency (e.g. means) or other basic estimates (e.g. regression coefficient) AND variation (e.g. standard deviation) or associated estimates of uncertainty (e.g. confidence intervals)
☐	☒	For null hypothesis testing, the test statistic (e.g. F, t, r) with confidence intervals, effect sizes, degrees of freedom and P value noted<br>*Give P values as exact values whenever suitable.*
☐	☒	For Bayesian analysis, information on the choice of priors and Markov chain Monte Carlo settings
☐	☒	For hierarchical and complex designs, identification of the appropriate level for tests and full reporting of outcomes
☐	☒	Estimates of effect sizes (e.g. Cohen's *d*, Pearson's *r*), indicating how they were calculated

Our web collection on statistics for biologists contains articles on many of the points above.

Software and code

Policy information about availability of computer code

Data collection	No software was used for data collection
Data analysis	1) A predicted (3D) structure of FcR1 was generated using ColabFold v.1.5.2 (https://github.com/sokrypton/ColabFold)<br>2) Protein structural visualization: ChimeraX v.1.6.1<br>3) Sequence alignment: MAFFT v.7.0<br>4) Maximum likelihood reference tree construction: RxML v.8.2<br>5) Analysis of electrophysiological data: OriginPro v. 2020<br>6) GLM was done using the glm function of the R stats package v. 4.2.1<br>7) Regression summaries were exported using jtools R package 2.2.0.112; Data analysis and visualization was done using the R tidyverse 113 and by using ggplot2 114 and ggtree.

For manuscripts utilizing custom algorithms or software that are central to the research but not yet described in published literature, software must be made available to editors and reviewers. We strongly encourage code deposition in a community repository (e.g. GitHub). See the Nature Portfolio guidelines for submitting code & software for further information.

Data

Policy information about availability of data

All manuscripts must include a data availability statement. This statement should provide the following information, where applicable:

- Accession codes, unique identifiers, or web links for publicly available datasets
- A description of any restrictions on data availability
- For clinical datasets or third party data, please ensure that the statement adheres to our policy

Fragilariopsis cylindrus genome sequence v.1.0 (CCMP 1102); Thalassiosira pseudonana genome sequence v.3.0 (CCMP1335); RNAseq data: E-MTAB-5429; Metatranscriptome data: E-MTAB-5024; All other data and biological materials are available from the authors upon request.

Research involving human participants, their data, or biological material

Policy information about studies with human participants or human data. See also policy information about sex, gender (identity/presentation), and sexual orientation and race, ethnicity and racism.

Reporting on sex and gender	N/A
Reporting on race, ethnicity, or other socially relevant groupings	N/A
Population characteristics	N/A
Recruitment	N/A
Ethics oversight	N/A

Note that full information on the approval of the study protocol must also be provided in the manuscript.

Field-specific reporting

Please select the one below that is the best fit for your research. If you are not sure, read the appropriate sections before making your selection.

- [x] Life sciences
- [ ] Behavioural & social sciences
- [ ] Ecological, evolutionary & environmental sciences

For a reference copy of the document with all sections, see nature.com/documents/nr-reporting-summary-flat.pdf

Life sciences study design

All studies must disclose on these points even when the disclosure is negative.

Sample size	All biological experiments were done at least in triplicate (N=3) for applying statistical tests. N=3 was chosen because it is the minimum number of replications for robust statistical tests. N > 3 would have been too resource demanding.
Data exclusions	No data were excluded.
Replication	All attempts at replication were successful. We replicated at least 3x times.
Randomization	Allocation was random.
Blinding	Biological experiments were impossible to conduct under conditions of blinding. Hence, there was no blinding.

Reporting for specific materials, systems and methods

We require information from authors about some types of materials, experimental systems and methods used in many studies. Here, indicate whether each material, system or method listed is relevant to your study. If you are not sure if a list item applies to your research, read the appropriate section before selecting a response.

Materials & experimental systems

n/a	Involved in the study	
☐	☒	Antibodies
☐	☒	Eukaryotic cell lines
☒	☐	Palaeontology and archaeology
☒	☐	Animals and other organisms
☒	☐	Clinical data
☒	☐	Dual use research of concern
☒	☐	Plants

Methods

n/a	Involved in the study	
☒	☐	ChIP-seq
☒	☐	Flow cytometry
☒	☐	MRI-based neuroimaging

Antibodies

Antibodies used	Bespoke antibodies were used. Hence, there is no catalog number. Supplier was Eurogentec, Seraing (Belgium). Polyclonal antibodies to FcR1/2 were raised against the antipeptides GELAKTGSPFNDAYR (extracellular loop between helix II and helix III, residues 104 - 118) and KSLALKNGNEETPLL (C-terminus, residues 275 - 289) and used to immunize two different rabbits each.
Validation	Validation was done by Eurogentec (using an ELISA). Immunoreactivity against the antigen was assessed by western blotting with the unpurified antiserum.

Eukaryotic cell lines

Policy information about cell lines and Sex and Gender in Research

Cell line source(s)	Custom-made diatom cell line over-expressing FcR1 in Thalassiosira pseudonana (CCMP1335) (Faktorova et al. 2020, Nat. Methods 17, 481-494)
Authentication	FcR1 in T. pseudonana (CCMP1335) was authenticated by sequencing the target locus in conjunction with the 18S locus in this diatom cell line.
Mycoplasma contamination	Cell lines were not tested for Mycoplasma contamination.
Commonly misidentified lines (See ICLAC register)	N/A
