# Supplementary figures and images for: Plastid-localized xanthorhodopsin increases diatom biomass and ecosystem productivity in iron-limited surface oceans
Source: Nat Microbiol. 2023 Oct 16;8(11):2050–66. doi: 10.1038/s41564-023-01498-5 (PMC10627834; doi:10.1038/s41564-023-01498-5)

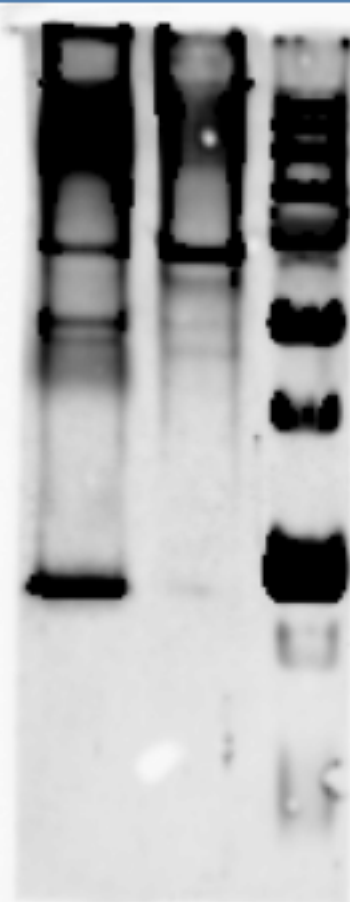

250 kDa  
150 kDa  
100 kDa  
75 kDa  
50 kDa  
37 kDa  
25 kDa  
20 kDa  
15 kDa

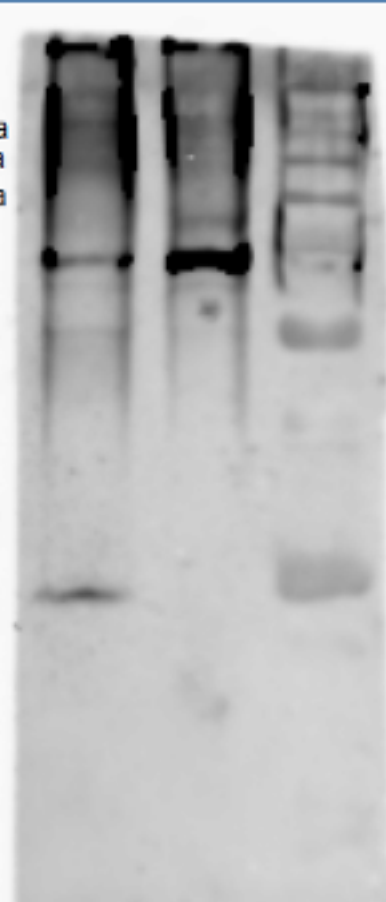

250 kDa  
150 kDa  
100 kDa  
75 kDa  
50 kDa  
37 kDa  
25 kDa  
20 kDa  
15 kDa

Supplement: Supplementary file 5 — Source data. [file 41564_2023_1498_MOESM5_ESM.zip › Strauss_Fig3c_SourceData.pdf]

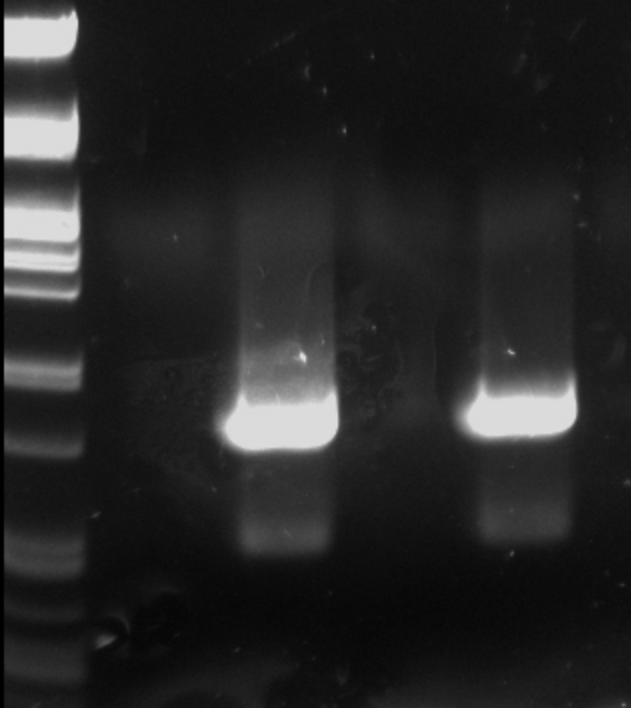

Supplement: Supplementary file 10 — Source data. [file 41564_2023_1498_MOESM10_ESM.pdf]
